# Supplementary material for: Dubosiella newyorkensis modulates immune tolerance in colitis via the L-lysine-activated AhR-IDO1-Kyn pathway
Source: Nat Commun. 2024 Feb 13;15:1333. doi: 10.1038/s41467-024-45636-x (PMC10864277; doi:10.1038/s41467-024-45636-x)
Supplement: Supplementary file 1 — Supplementary Information [file 41467_2024_45636_MOESM1_ESM.pdf]

# Supplementary information for

# *Dubosiella newyorkensis* modulates immune tolerance in colitis through L-lysine-activated AhR-IDO1-Kyn metabolic circuitry

**Yanan Zhang, Shuyu Tu, Xingwei Ji, Jianan Wu, Jinxin Meng, Jinsong Gao, Xian Shao, Shuai Shi, Gan Wang, Jingjing Qiu, Zhuobiao Zhang, Chengang Hua, Ziyi Zhang, Shuxian Chen, Li Zhang and Shu Jeffrey Zhu**

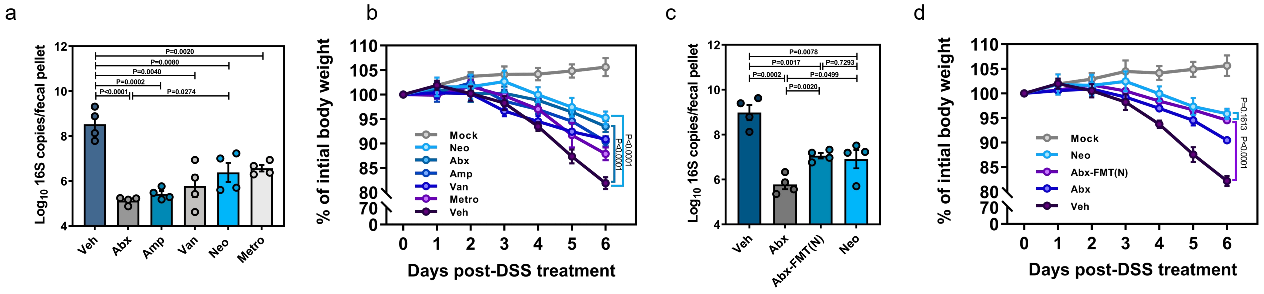


**Supplementary Fig. 1 Neomycin confers protection from DSS-induced colitis.** 16S rDNA copies per fecal pellet prior to DSS administration (n=4) (a) and daily body weight during DSS treatment (b) were determined in mice treated with vehicle, different single antibiotics (mock, Veh, Neo, n=6; Amp, Van, Metro, n=4) or in combination (Abx, n=4). (c) 16S rDNA copies per fecal pellet prior to DSS administration (n=4) and (d) daily body weight during DSS treatment in Abx-treated mice with or without fecal microbiota transplantation (FMT) from Neo-treated mice (mock, Veh, n=5; Neo, Abx, FMT, n=4). Results are representative of data generated in at least two independent experiments and are expressed as the mean ± SEM. Unpaired 2-tailed Student's *t*-tests (a and c) and one-way ANOVA (b and d) were used for statistical analysis.

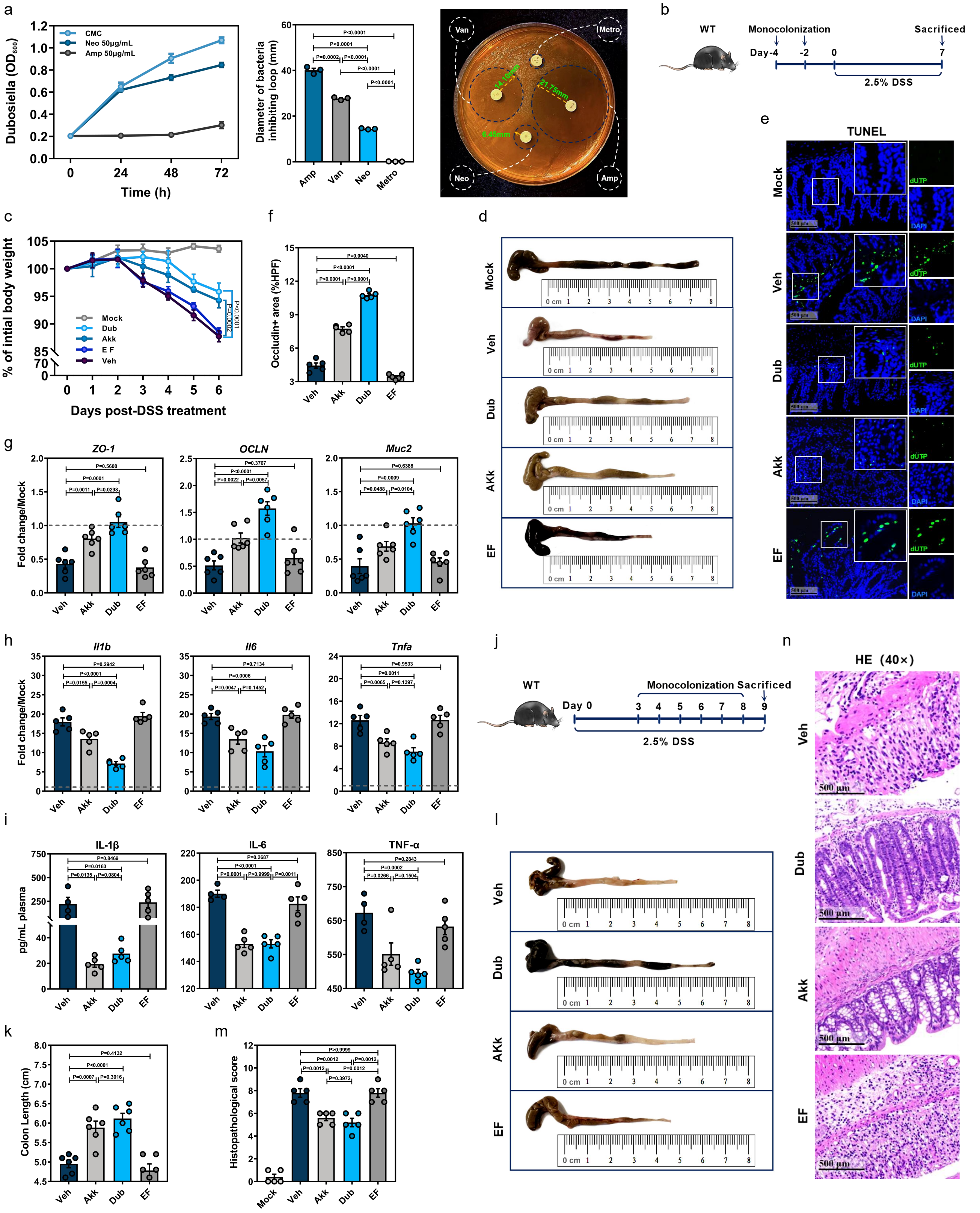


**Supplementary Fig. 2 Oral administration of *D. newyorkensis* reduces susceptibility to DSS-induced colitis.** (a) *D. newyorkensis* (Dub) was grown for 72 h in chopped meat carbohydrate (CMC) media in the presence of 50 μg/mL neomycin (Neo) or ampicillin (Amp) or without antibiotics (CMC). $OD_{600}$ was measured

every 24 h. Diameter of bacteria inhibiting loop was determined after culture of Dub in the presence of Amp, Van, Neo or Metro (n=3). (b-i) Conventional wild-type C57BL/6J mice were colonized with $10^9$ CFU of *D. newyorkensis* (Dub), *A. muciniphila* (Akk), *E. faecalis* (EF) or vehicle (Veh) twice with 2-day break in between, then treated with DSS as previously described. (c) Body weight was monitored daily from D0 to D6 post-DSS treatment (n=4). At D7 post-DSS administration, colon samples were collected to determine colon length (d), colonic cell apoptosis by TUNEL staining (e) and Occludin-positive area by IFA (n=5) (f). The expression of *ZO-1*, *OCLN* and *Muc2* in colonic intestinal epithelial cells (cIECs) (n=6) (g), and the level of *Il1b*, *Il6* and *Tnfa* in colonic lamina propria cells (cLPs) (h) were determined by qRT-PCR (n=5). Serum levels of IL-1β, IL-6 and TNF-α were examined by ELISA (Veh, n=4; Akk, Dub, EF, n=5) (i). (j-n) Conventional WT mice (n=6) were treated with 2.5% DSS in drinking water for 3 days and gavaged with $10^9$ CFU of Dub, Akk or EF daily from the 3rd day to the $8^{th}$ day in addition to 2.5% DSS. At D9 post-DSS treatment, colon length (n=6) (k and l) and histopathological score by HE staining (n=5) (m and n) were examined. Results are representative of data generated in at least two independent experiments and are expressed as mean ± SEM. Unpaired 2-tailed Student's *t*-tests and one-way ANOVA (c) were used for statistical analysis.

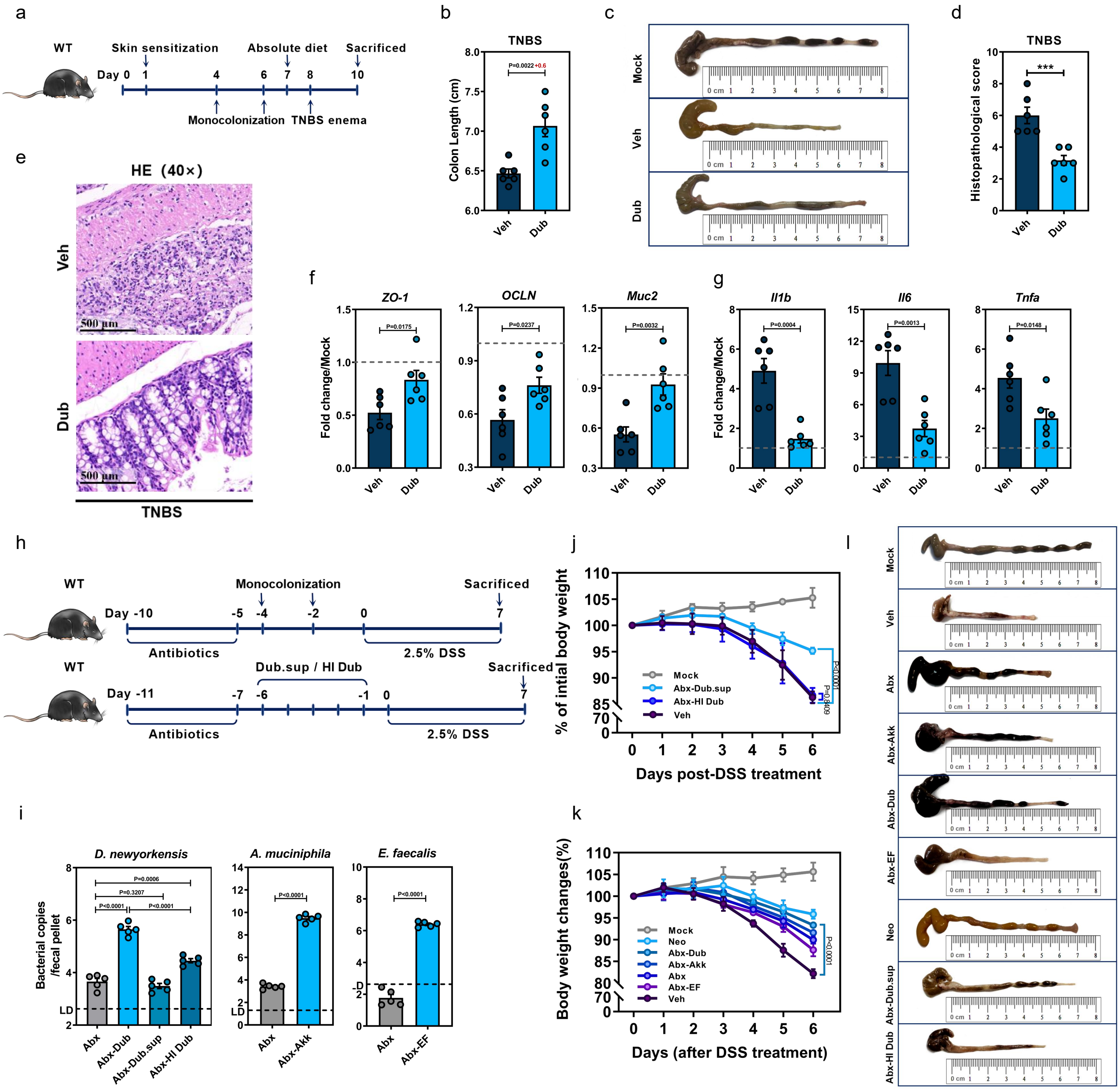

**Supplementary Fig. 3 Oral administration of *D. newyorkensis* reduces susceptibility to chemically induced colitis.** (a) Schematic showing administration schedule for TNBS in *D. newyorkensis* (Dub)-colonized ($10^9$ CFU) or noncolonized mice. Measurement of colon length (b and c) and histopathological evaluation (d) by HE staining (e) was performed. The expression of *ZO-1*, *OCLN* and *Muc2* in colonic intestinal epithelial cells (cIECs) (f), and the level of *Il1b*, *Il6* and *Tnfa* in the colonic lamina propria cells (cLPs) (g) was determined by qRT-PCR (n=6). (h) Schematic showing oral gavage in Abx-treated mice with different bacterial strains ($10^9$ CFU of Dub, *A. muciniphila* [Akk], or *E. faecalis* [EF]), or treatment with heat-inactivated (HI) Dub or Dub culture supernatant (Dub.sup) before DSS exposure. (i) qPCR determination of

Dub, Akk or EF genomic copies in fecal samples from Abx-treated mice with or without bacterial colonization prior to DSS administration (n=5). Body weight of Abx-mice treated with HI Dub, Dub.sup or Veh (mock, n=4; Veh, Abx-Dub.sup, n=6, Abx-HI Dub, n=5) (j) and body weight of Abx-treated mice colonized with $10^9$ CFU of different bacterial strains (mock, Veh, n=5; Neo, Abx-Dub, Abx-Akk, Abx-EF, n=4) (k) were documented every day from D0 to D6 post-DSS treatment. (l) Colon length of Abx-treated mice colonized with $10^9$ CFU of different bacterial strains, or treated with HI Dub or Dub.sup was measured at D7 post-DSS treatment (n=5). Results are representative of data generated in at least two independent experiments and are expressed as mean $\pm$ SEM. Unpaired 2-tailed Student's *t*-tests and one-way ANOVA (j and k) were used for statistical analysis.

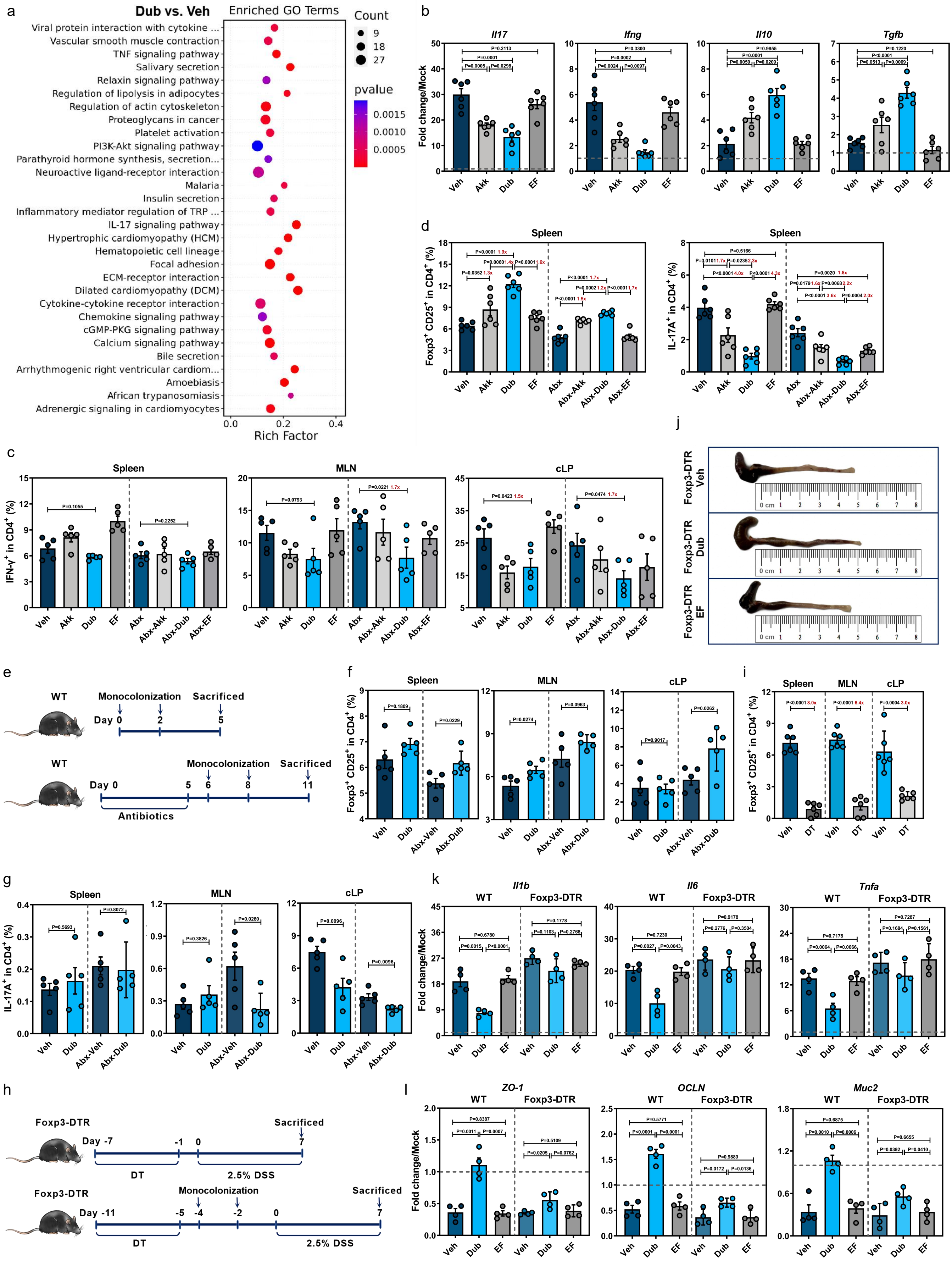
a
Dub vs. Veh
Enriched GO Terms
Viral protein interaction with cytokine ...
Vascular smooth muscle contraction
TNF signaling pathway
Salivary secretion
Relaxin signaling pathway
Regulation of lipolysis in adipocytes
Regulation of actin cytoskeleton
Proteoglycans in cancer
Platelet activation
PI3K-Akt signaling pathway
Parathyroid hormone synthesis, secretion...
Neuroactive ligand-receptor interaction
Malaria
Insulin secretion
Inflammatory mediator regulation of TRP ...
IL-17 signaling pathway
Hypertrophic cardiomyopathy (HCM)
Hematopoietic cell lineage
Focal adhesion
ECM-receptor interaction
Dilated cardiomyopathy (DCM)
Cytokine-cytokine receptor interaction
Chemokine signaling pathway
cGMP-PKG signaling pathway
Calcium signaling pathway
Bile secretion
Arrhythmogenic right ventricular cardiom...
Amoebiasis
African trypanosomiasis
Adrenergic signaling in cardiomyocytes
Rich Factor
Count
9
18
27
pvalue
0.0015
0.0010
0.0005
b
Il17
Ifng
Il10
Tgfb
Fold change/Mock
Veh
Akk
Dub
EF
c
Spleen
MLN
cLP
IFN-γ+ in CD4+ (%)
Abx
Abx-Akk
Abx-Dub
Abx-EF
d
Spleen
Foxp3+ CD25+ in CD4+ (%)
IL-17A+ in CD4+ (%)
e
WT
Monocolonization
Sacrificed
Day
Antibiotics
f
Abx-Veh
g
h
Foxp3-DTR
DT
2.5% DSS
i
DT
j
Foxp3-DTR Veh
Foxp3-DTR Dub
Foxp3-DTR EF
k
Il1b
Il6
Tnfa
WT
Foxp3-DTR
l
ZO-1
OCLN
Muc2

**Supplementary Fig. 4 *D. newyorkensis* mitigates DSS-induced colitis by modulating CD25⁺Foxp3⁺ Tregs.** (a) KEGG analysis of differentially expressed genes in colon samples between vehicle (Veh)-treated (n=3) and *D. newyorkensis* (Dub)-colonized wild-type C57BL/6J mice (n=4) at D7 post-DSS treatment. (b) qRT-PCR of indicated cytokines in colonic lamina propria mononuclear cells from conventional WT mice colonized with $10^9$ CFU of Dub, *A. muciniphila* (Akk), *E. faecalis* (EF), or Veh (n=6). (c) IFNγ$^+$CD4$^+$ T cells in spleen, mesenteric lymph nodes (MLN) and colonic lamina propria (cLP) of conventional or Abx-treated WT mice colonized with $10^9$ CFU of Dub, Akk or EF (n=5). (d) Splenic CD25$^+$Foxp3$^+$Tregs and IL-17$^+$ CD4$^+$ T cells in conventional or Abx-treated WT mice colonized with $10^9$ CFU of Dub, Akk or EF (n=6). (e-g) Conventional or Abx-treated WT mice were colonized with $10^9$ CFU of Dub or Veh twice with a 48-h interval without DSS exposure (n=5). CD25$^+$Foxp3$^+$Tregs (f) and IL-17$^+$ CD4$^+$ T cells (g) in spleen, MLN or cLP of conventional or Abx-treated WT mice colonized with $10^9$ CFU of Dub or Veh at D3 following the second bacterial colonization. (h-l) Conventional WT or diphtheria toxin (DT)-injected Foxp3-DTR mice were colonized with $10^9$ CFU of Dub or EF. Depletion of CD25$^+$Foxp3$^+$Tregs in the spleen, MLN or cLP of Foxp3-DTR mice treated with or without DT was verified by flow cytometry (i) prior to DSS exposure (n=6). At D7 post-DSS administration, colon length (n=5) (j) was measured and the expression levels of *Il1b*, *Il6* and *Tnfa* in cLPs (k) and *ZO-1*, *OCLN* and *Muc2* in colonic intestinal epithelial cells (IECs) (l) were determined by qRT-PCR (n=4). Results are representative of data generated in at least two independent experiments and are expressed as mean ± SEM. Unpaired 2-tailed Student's *t*-tests were used for statistical analysis.

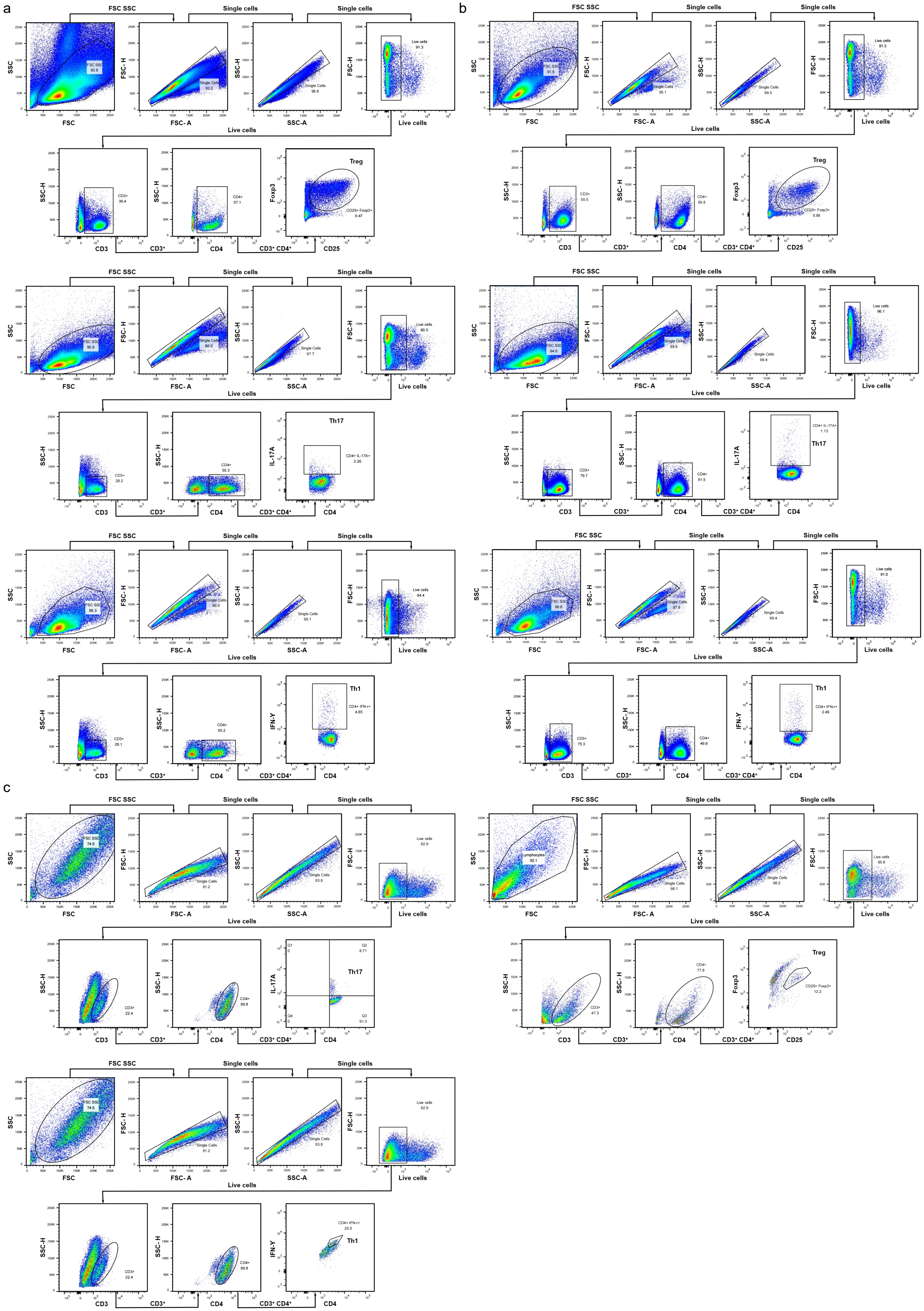
a
b
c
FSC SSC
Single cells
Live cells
CD3+
CD3+ CD4+
Treg
Th17
Th1
SSC
FSC
FSC- H
FSC- A
SSC-H
SSC-A
SSC- H
CD3
CD4
CD25
Foxp3
IL-17A
IFN-Y

**Supplementary Fig. 5 Flow cytometry gating scheme of $CD25^+Foxp3^+$Tregs, IL-$17^+$ $CD4^+$ T cells and IFNγ$^+$CD$4^+$ T cells in different tissues.** Representative flow cytometry plots showing gating strategy for the indicated T cell subsets from spleen (a), mesenteric lymph nodes (MLN) (b) and colonic lamina propria (cLP) (c).

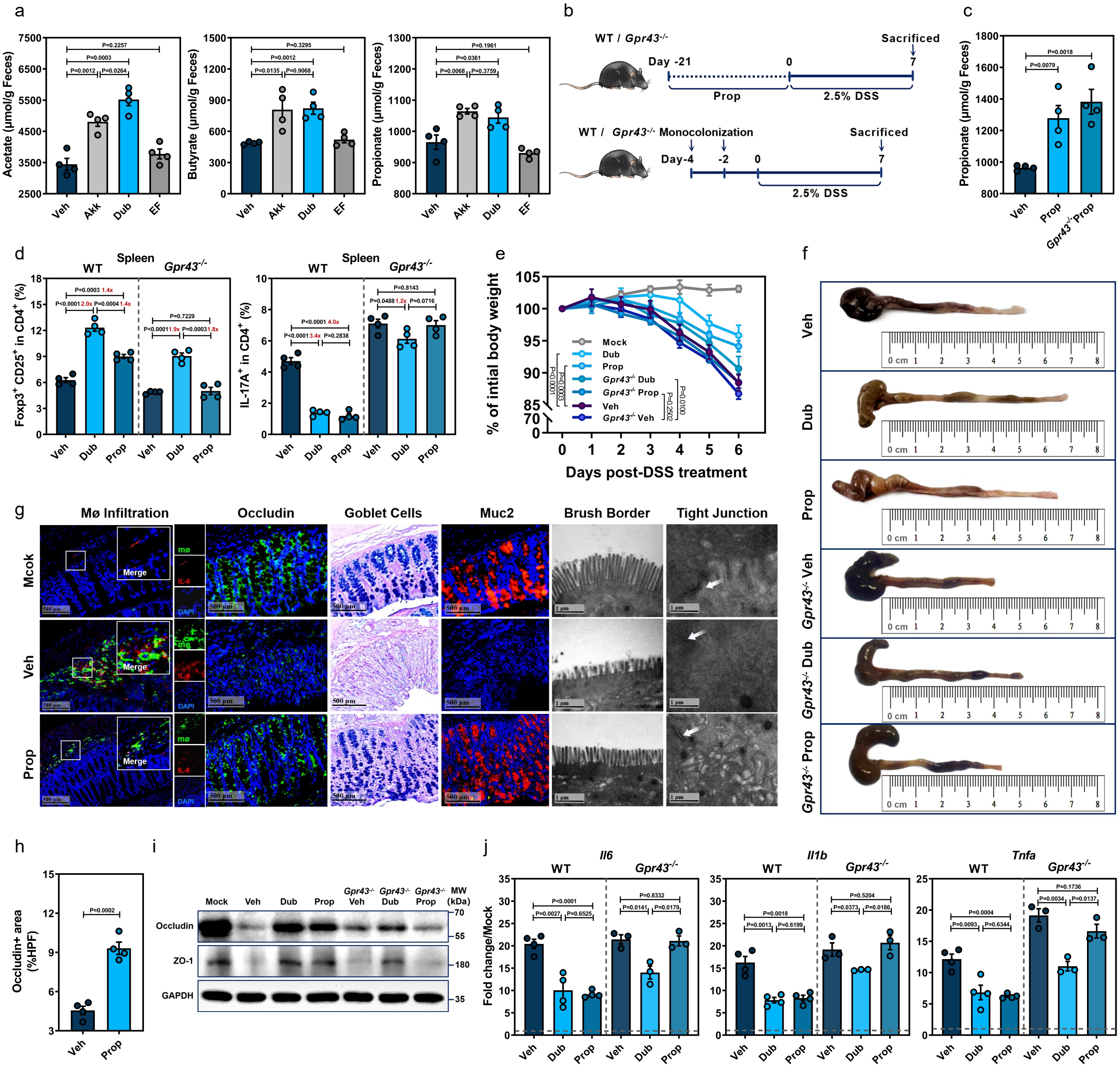


**Supplementary Fig. 6 Oral administration of propionate suppresses DSS-induced colon injury by modulating CD25⁺Foxp3⁺ Tregs in a GPR43-dependent manner.** Concentration of short-chain fatty acids (SCFA) were determined in fecal samples (a) collected from conventional wild-type C57BL/6J mice (WT) colonized with $10^9$ CFU of *D. newyorkensis* (Dub), *A. muciniphila* (Akk), *E. faecalis* (EF), or vehicle (Veh) before DSS administration (n=4). (b-j) Conventional WT or *Gpr43*$^{-/-}$ mice were colonized with Dub ($10^9$ CFU) twice or administered propionate (Prop, 150 mM) in drinking water for 3 weeks and concentration of Prop in fecal samples was determined (c) before DSS administration (n=4). (d) Splenic CD25+Foxp3+Tregs and IL-17+ CD4+ T cells in Dub-colonized, Prop- or Veh-treated conventional WT or Gpr43$^{-/-}$ mice at D7 post-DSS administration (n=4). Body weight (e) was monitored daily from D0 to D6

post-DSS administration (n=4). Colon length (f), macrophage infiltration and parameters regarding mucosal barrier conditions described in Fig. 2c-d were determined in Prop- or Veh-treated conventional WT mice (n=4) (g-i). (j) Expression of *Il1b*, *Il6* and *Tnfa* in the colonic lamina propria of Dub-colonized or Prop- or Veh-treated conventional WT or *Gpr43*$^{-/-}$ mice was determined by qRT-PCR (WT, n=4; *Gpr43*$^{-/-}$, n=3). Results are representative of data generated in at least two independent experiments and are expressed as mean ± SEM. Unpaired 2-tailed Student's *t*-tests and one-way ANOVA (e) were used for statistical analysis.

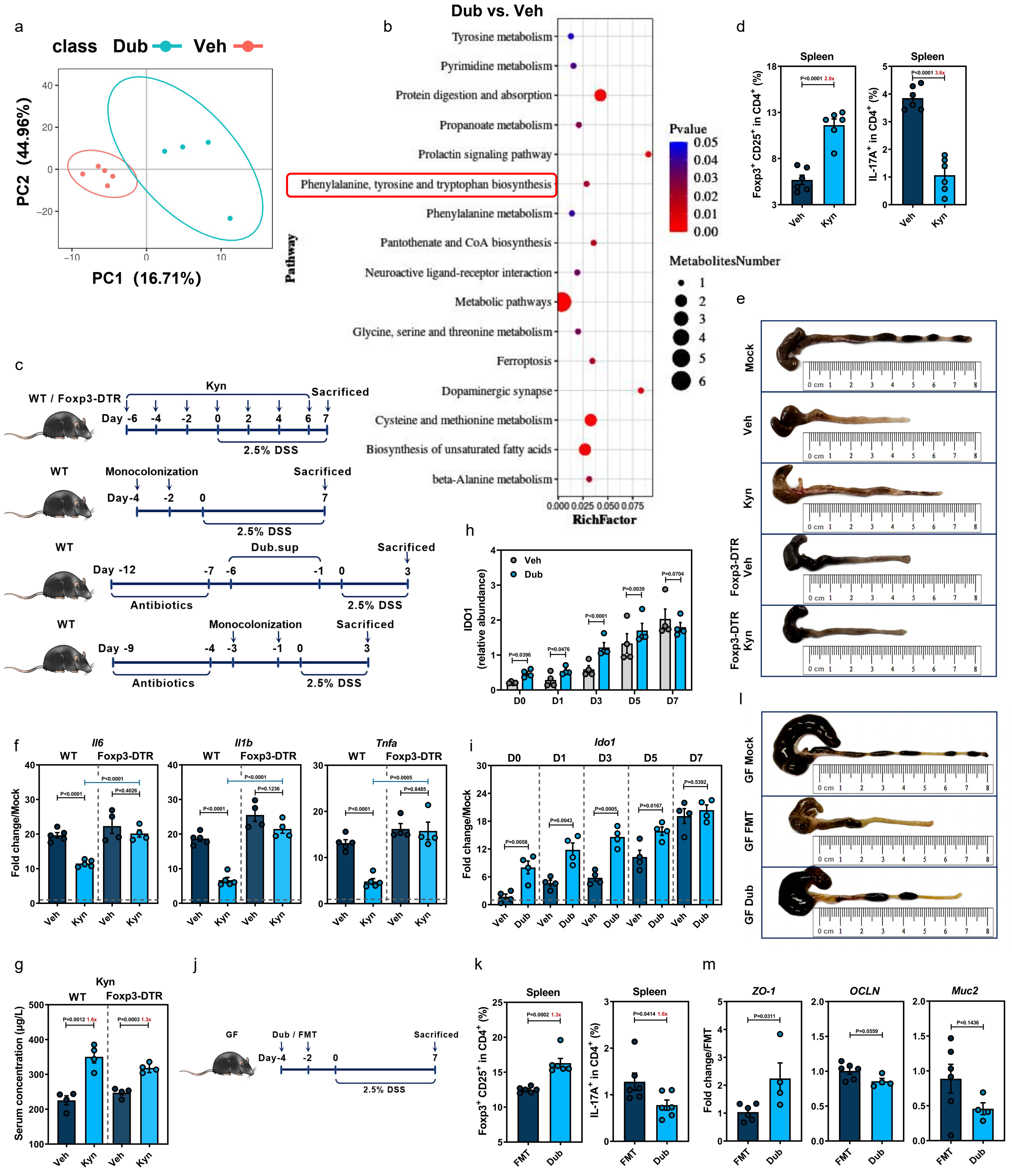

**Supplementary Fig. 7 Colonization by *D. newyorkensis* enhances Trp metabolism in dendritic cells to ameliorate DSS-induced inflammation in a Treg-dependent manner.** PCA (a) and KEGG analysis (b) of differentially expressed metabolites in colon samples from conventional wild-type C57BL/6J mice (WT) colonized with $10^9$ CFU of *D. newyorkensis* (Dub, n=5) or vehicle (Veh, n=4) at D7 post-DSS administration. (c) Study schematics showing kynurenine (Kyn) protection study (top), detection of colonic IDO1

expression in Dub-colonized and noncolonized conventional WT mice during the course of DSS administration, and detection of IDO1 expression in colon-specific cell types of Dub.sup-treated or Dub-colonized Abx-treated mice in the presence of DSS. (e-g) Conventional WT or DT-treated Foxp3-DTR mice (n=6) were treated with Kyn and subjected to DSS (d) Splenic $CD25^+Foxp3^+$Tregs and IL-$17^+$ $CD4^+$ T cells in Veh- or Kyn-treated conventional WT mice at D7 post-DSS administration. Comparison of colon length (n=4) (e) and proinflammatory cytokine expression in colonic lamina propria (f) between Kyn-treated and untreated WT or Foxp3-DTR mice (WT, n=5; Foxp3-DTR, n=4). (g) Serum Kyn concentrations of both mouse strains were detected before exposure to DSS (n=4). (h) Quantification of proteins in Fig. 4h. The protein levels of IDO1 were normalized to those of GAPDH (n=4). (i) Kinetics of colonic *Ido1* expression in Dub-colonized and noncolonized conventional WT mice during the course of DSS administration by qRT-PCR (n=4). (j-m) Germ-free (GF) mice received fecal microbiota transplantation (FMT) from conventional WT mice or were colonized with Dub ($10^6$ CFU) before DSS administration. Splenic $CD25^+Foxp3^+$Tregs and IL-$17^+$ $CD4^+$ T cells (n=6) (k), colon length (l) and expression of *ZO-1*, *OCLN* and *Muc2* in colonic intestinal epithelial cells of Dub-colonized or FMT-treated GF mice at D7 post-DSS administration (FMT, n=6; Dub, n=4) (m). Results are representative of data generated in at least two independent experiments and are expressed as mean ± SEM. Unpaired 2-tailed Student's *t*-tests were used for statistical analysis.

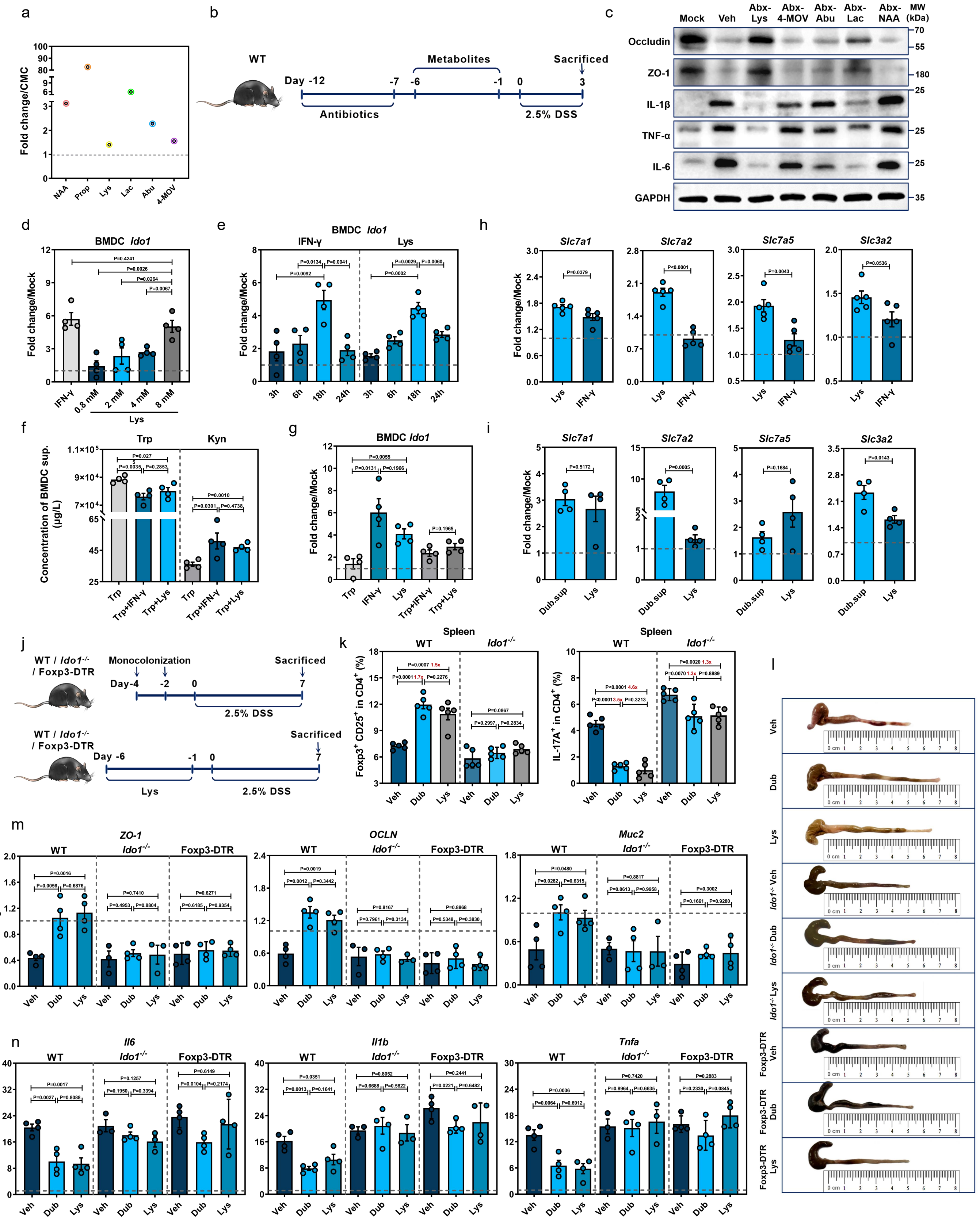

**Supplementary Fig. 8 *D. newyorkensis*-derived Lys augments IDO1-mediated Trp metabolism in dendritic cells to modulate Treg-dependent immune tolerance.** (a) Relative abundance of positive or

negative metabolites in Dub cultured supernatant (Dub.sup). (b) Schematics showing Abx-treated wild-type C57BL/6J mice (WT) treated with metabolites and subjected to DSS administration. At D7 post-DSS treatment, the protein expression of ZO-1, Occludin and proinflammatory cytokines IL-1β, IL-6 and TNF-α (c) in colon samples were determined by western blot. (d) *Ido1* expression in mouse bone marrow-derived dendritic cells (BMDCs) treated with different doses of Lys (n=4). (e) *Ido1* expression in mouse BMDCs at indicated time points post Lys or IFN-γ treatment (n=4). Concentration of Trp and kynurenine (Kyn) in the culture SUP (f) and *Ido1* expression (g) of mouse BMDCs treated with Lys or IFN-γ in the presence of Trp (n=4). Expression of Lys transporters SLC7A1 and SLC7A2, and Trp transporters SLC7A5 and SLC3A2 in Lys- or IFN-γ-treated BMDCs extracted from conventional WT mice (n=5) (h), and in the Lys- or Dub.sup-treated BMDCs extracted from GF mice (n=4) (i). For the experiments shown in (d-i) above, the following were used: Lys (8 mM), IFN-γ (20 ng/mL) and Trp (100 mg/L). (j) Study schematics showing conventional WT, DT-treated Foxp3-DTR or *Ido1*$^{-/-}$ mice colonized with $10^9$ CFU of Dub or treated with 20 mg/kg Lys and subjected to DSS administration. At D7 post-DSS administration, splenic $CD25^+Foxp3^+$Tregs and IL-17$^+$ $CD4^+$ T cells (n=5) (k), colon length (n=4) (l), expression of *ZO-1*, *OCLN* and *Muc2* in colonic intestinal epithelial cells (m), and expression of *Il1b*, *Il6* and *Tnfa* in the colonic lamina propria (n) were determined (WT, Foxp3-DTR, n=4; *Ido1*$^{-/-}$ Veh, *Ido1*$^{-/-}$ Lys, n=3; *Ido1*$^{-/-}$ Dub, n=4). Plotted data represent the mean ± SEM. Unpaired 2-tailed Student's *t*-tests were used for statistical analysis.

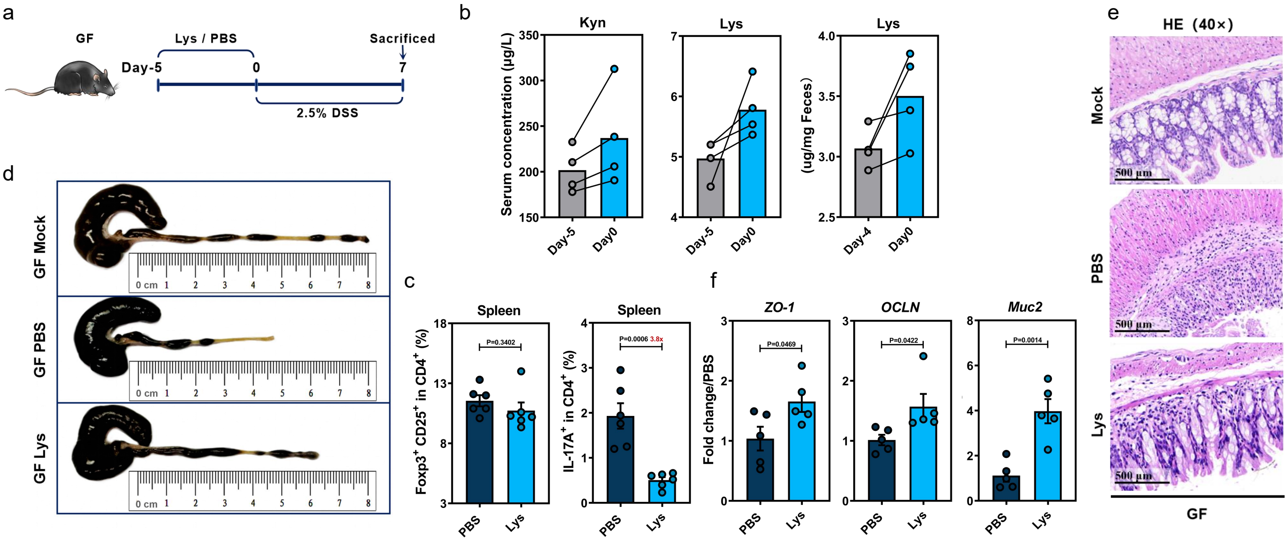


**Supplementary Fig. 9 Lys augments Trp metabolism to modulate Treg-dependent immune tolerance in GF mice.** (a) Study schematic showing germ-free (GF) mice treated with 20 mg/kg Lys and subjected to DSS administration. (b) Comparison of kynurenine (Kyn) and Lys concentrations in the sera of GF mice before and after Lys treatment (n=4); Lys concentration in the feces of GF mice before and after *D. newyorkensis* (Dub) colonization (see Fig. S7j). At D7 post-DSS administration, splenic $CD25^+Foxp3^+$Tregs and IL-$17^+$ $CD4^+$ T cells (n=6) (c), colon length (n=5) (d), histopathological evaluation by HE staining (e) and expression of *ZO-1, OCLN* and *Muc2* in colonic intestinal epithelial cells (n=5) (f) were determined. Results are representative of data generated in at least two independent experiments and are expressed as mean ± SEM. Unpaired 2-tailed Student's *t*-tests were used for statistical analysis.

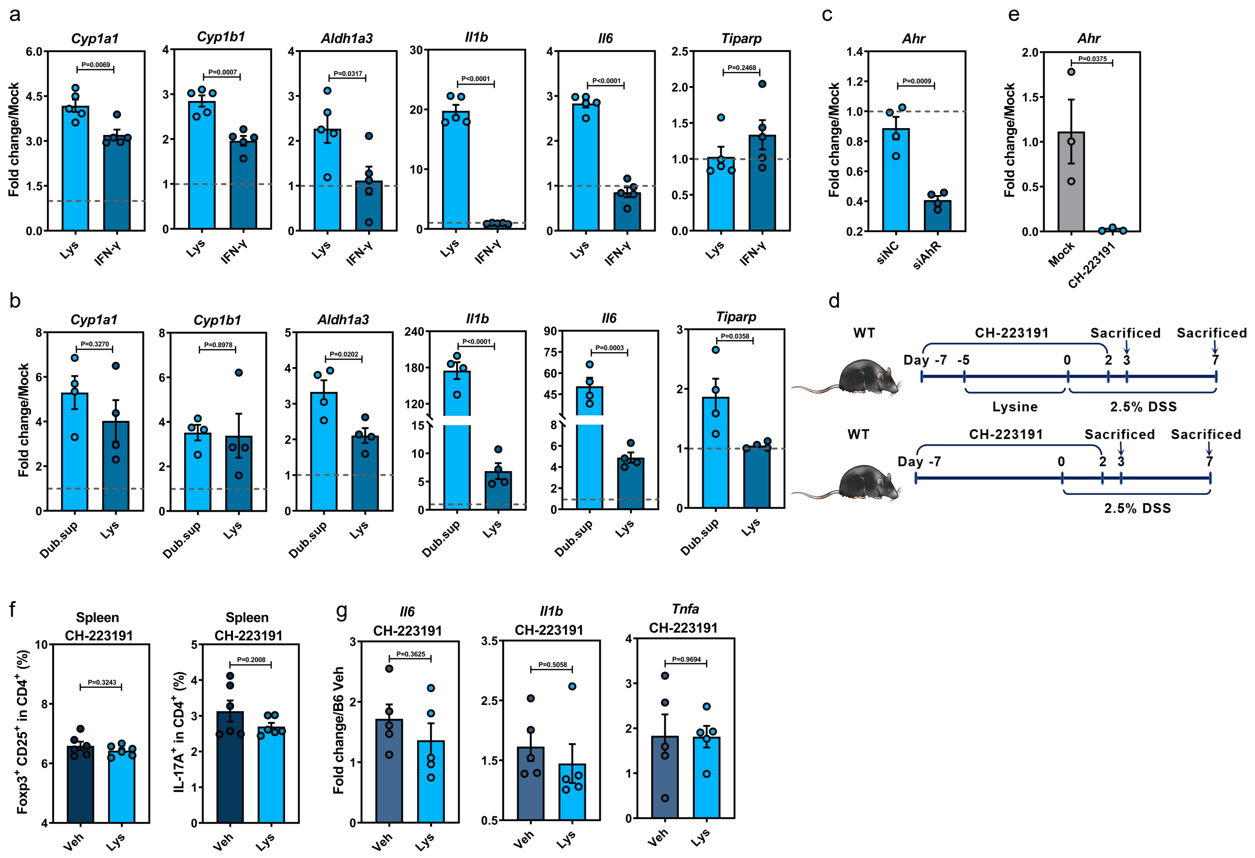


**Supplementary Fig. 10 Lys activates AhR in DCs to induce Treg-mediated immunosuppression in DSS-induced colitis.** Expression of AhR target genes in Lys- or IFN-γ-treated BMDCs extracted from conventional wild-type C57BL/6J mice (WT, n=5) (a), and in the Lys- or Dub.sup-treated BMDCs extracted from GF mice at 6 h post-treatment (n=4) (b). For the experiments shown in (a-b) above, the following were used: Lys (8 mM) and IFN-γ (20 ng/mL). (c) qRT-PCR of *AhR* mRNA in WT mouse BMDCs transfected with negative control (siNC) or AhR-specific small interfering RNA (siAhR) at 24 h (n=4). (d) Study schematics showing Lys-treated (20 mg/kg) conventional WT mice with or without 10 mg/kg CH-223191 intervention before DSS exposure. (e) qRT-PCR of *AhR* mRNA in colonic lamina propria mononuclear cells from CH-223191-treated or untreated conventional WT mice (n=3). Splenic $CD25^+Foxp3^+$Tregs and IL-$17^+$ $CD4^+$ T cells (n=6) (f) and expression of *Il1b*, *Il6* and *Tnfa* in the colonic lamina propria were determined (n=5) (g). Results are representative of data generated in at least two independent experiments and are expressed as mean ± SEM. Unpaired 2-tailed Student's *t*-tests were used for statistical analysis.

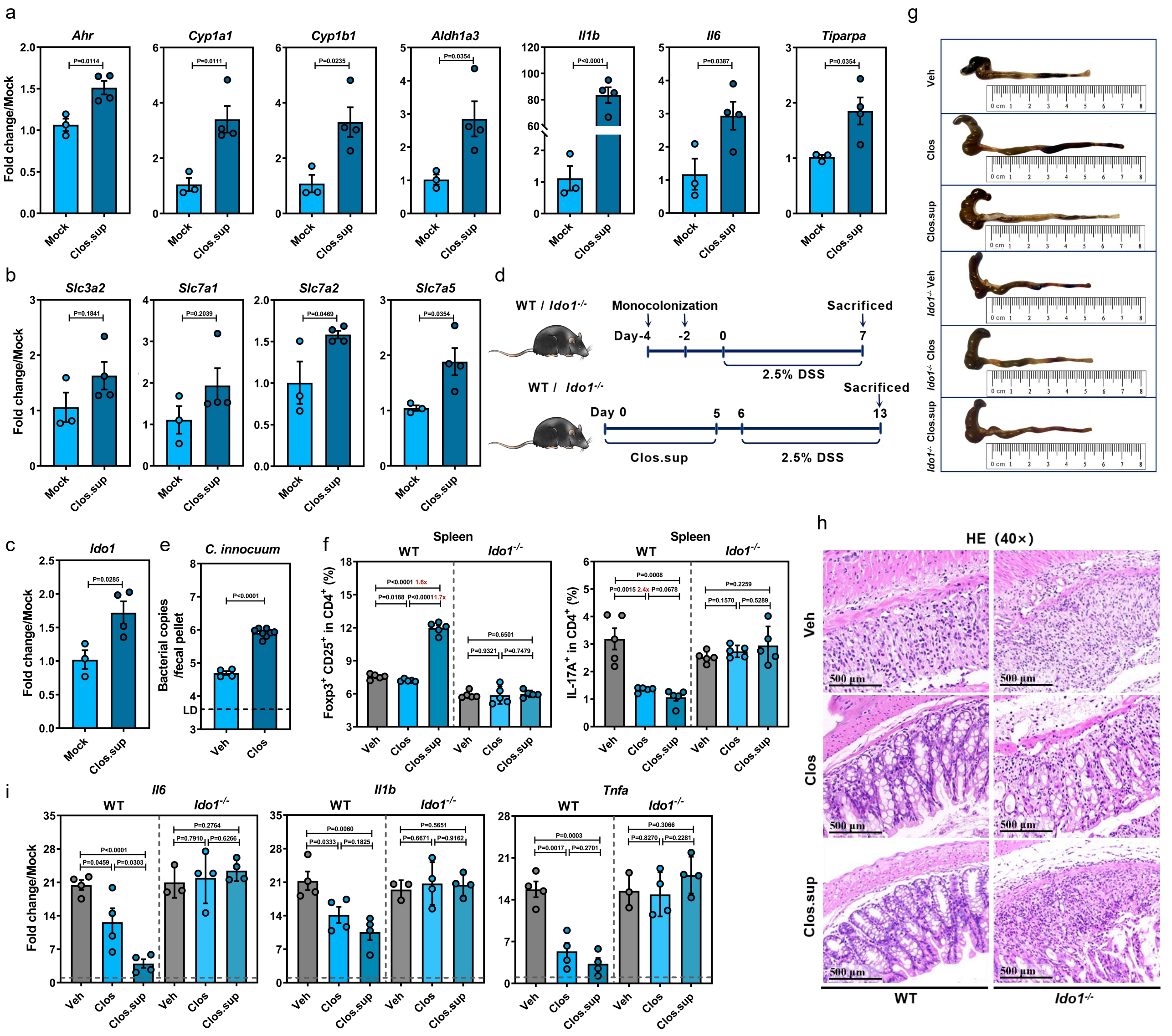

**Supplementary Fig. 11 *C. innocuum* is the homologue of *D. newyorkensis* in mouse models, protecting against DSS-induced colitis in an IDO1-dependent manner.** (a-c) Bone marrow-derived dendritic cells from germ-free (GF) mice were treated with *C. innocuum* culture supernatant (Clos.sup) (mock, n=3; Clos.sup, n=4). Expression of *Ahr* and target genes (*Cyp1a1*, *Cyp1b1*, *Aldh1a3, Il1b, Il6, Tiparpa*) (a), and genes encoding Lys and Trp transporters (*Slc3a2*, *Slc7a1*, *Slc7a2*, and *Slc7a5*) (b) were quantified at 6 h post-treatment, and *Ido1* expression was quantified at 18 h post-treatment by qRT-PCR (c). (d) Study schematics showing conventional wild-type C57BL/6J mice (WT) or *Ido1*$^{-/-}$ mice (n=5-6) colonized with *C. innocuum* ($10^9$ CFU, Clos) twice or administrated with *C. innocuum* supernatant (Clos.sup) for 5 days and then exposed to DSS treatment. (e) qPCR quantification of Clos genomic copies in fecal samples from Clos-colonized or noncolonized WT mice before DSS treatment (n=4). (f-i) At D7 post-DSS

administration, splenic $CD25^+Foxp3^+$Tregs and IL-$17^+$ $CD4^+$ T cells (n=5) (f), colon length (n=4) (g), histopathological evaluation by HE staining (h) and expression of *Il1b*, *Il6* and *Tnfa* in the colonic lamina propria were determined (WT, n=4; *Ido1*$^{-/-}$ Veh, n=3; *Ido1*$^{-/-}$ Clos, *Ido1*$^{-/-}$ Clos.sup, n=4) (i). Results are representative of data generated in at least two independent experiments and are expressed as mean ± SEM. Unpaired 2-tailed Student's *t*-tests were used for statistical analysis.
