# Supplementary material for: Dubosiella newyorkensis modulates immune tolerance in colitis via the L-lysine-activated AhR-IDO1-Kyn pathway
Source: Nat Commun. 2024 Feb 13;15:1333. doi: 10.1038/s41467-024-45636-x (PMC10864277; doi:10.1038/s41467-024-45636-x)
Supplement: Supplementary file 3 — Reporting Summary [file 41467_2024_45636_MOESM3_ESM.pdf]

nature portfolio

Corresponding author(s): Shu Jeffrey Zhu

Last updated by author(s): Jan 22, 2024

Reporting Summary

Nature Portfolio wishes to improve the reproducibility of the work that we publish. This form provides structure for consistency and transparency in reporting. For further information on Nature Portfolio policies, see our Editorial Policies and the Editorial Policy Checklist.

Statistics

For all statistical analyses, confirm that the following items are present in the figure legend, table legend, main text, or Methods section.

n/a	Confirmed	
[ ]	[x]	The exact sample size (*n*) for each experimental group/condition, given as a discrete number and unit of measurement
[ ]	[x]	A statement on whether measurements were taken from distinct samples or whether the same sample was measured repeatedly
[ ]	[x]	The statistical test(s) used AND whether they are one- or two-sided<br>*Only common tests should be described solely by name; describe more complex techniques in the Methods section.*
[ ]	[x]	A description of all covariates tested
[ ]	[x]	A description of any assumptions or corrections, such as tests of normality and adjustment for multiple comparisons
[ ]	[x]	A full description of the statistical parameters including central tendency (e.g. means) or other basic estimates (e.g. regression coefficient) AND variation (e.g. standard deviation) or associated estimates of uncertainty (e.g. confidence intervals)
[ ]	[x]	For null hypothesis testing, the test statistic (e.g. *F*, *t*, *r*) with confidence intervals, effect sizes, degrees of freedom and *P* value noted<br>*Give P values as exact values whenever suitable.*
[x]	[ ]	For Bayesian analysis, information on the choice of priors and Markov chain Monte Carlo settings
[x]	[ ]	For hierarchical and complex designs, identification of the appropriate level for tests and full reporting of outcomes
[x]	[ ]	Estimates of effect sizes (e.g. Cohen's *d*, Pearson's *r*), indicating how they were calculated

Our web collection on statistics for biologists contains articles on many of the points above.

Software and code

Policy information about availability of computer code

Data collection	No software was used
Data analysis	CaseViewer 2.4 and Image J were used to analyze H. E and IFA stained slides. Flowjo V10.0 was used to analyze flow cytometric data. GraphPad Prism V8.0 was used to generate graphs and for performing statistical analysis.

For manuscripts utilizing custom algorithms or software that are central to the research but not yet described in published literature, software must be made available to editors and reviewers. We strongly encourage code deposition in a community repository (e.g. GitHub). See the Nature Portfolio guidelines for submitting code & software for further information.

Data

Policy information about availability of data

All manuscripts must include a data availability statement. This statement should provide the following information, where applicable:

- Accession codes, unique identifiers, or web links for publicly available datasets
- A description of any restrictions on data availability
- For clinical datasets or third party data, please ensure that the statement adheres to our policy

16S rRNA gene sequence data are available in the Sequence Read Archive (SRA) under BioProject accession PRJNA1013230. RNA-seq data are available in the SRA under BioProject accession PRJNA1012836. The mouse colon homogenates and bacterial cell culture supernatants metabolome data reported in this study have been deposited in the NGDC OMIX database (OMIX ID: OMIX005711, https://ngdc.cncb.ac.cn/omix/release/OMIX005711; OMIX005712, https://ngdc.cncb.ac.cn/

omix/release/OMIX005712). All other data supporting the conclusions of this study are available in the paper and supplemental materials. Source data are provided with this paper.

Research involving human participants, their data, or biological material

Policy information about studies with human participants or human data. See also policy information about sex, gender (identity/presentation), and sexual orientation and race, ethnicity and racism.

Reporting on sex and gender	Seven healthy individuals (3 males and 4 females) aged from 22 to 41 were included in this study.
Reporting on race, ethnicity, or other socially relevant groupings	Not applicable.
Population characteristics	Peripheral blood specimens were collected from these healthy human participants and and peripheral blood mononuclear cells (PBMCs) were extracted.
Recruitment	Healthy individuals admitted to the hospital for healthy examination were recruited as healthy donors in the study.
Ethics oversight	This study was performed with the approval of the Ethical Committee of The First Affiliated Hospital of Guangdong Pharmaceutical University (Permit Number: 20210221). All participants provided written informed consent for sample collection and subsequent analyses.

Note that full information on the approval of the study protocol must also be provided in the manuscript.

Field-specific reporting

Please select the one below that is the best fit for your research. If you are not sure, read the appropriate sections before making your selection.

[x] Life sciences [ ] Behavioural & social sciences [ ] Ecological, evolutionary & environmental sciences

For a reference copy of the document with all sections, see nature.com/documents/nr-reporting-summary-flat.pdf

Life sciences study design

All studies must disclose on these points even when the disclosure is negative.

Sample size	No statistical methods were used to determine sample size. The sample sizes were selected based on previous experience and published literature. For in vitro and in vivo experiments, the minimum of sample size was 3 in all the cases. The sample size is stated in the figure legends.
Data exclusions	No data exclusion was performed.
Replication	All the experiments were replicated. The number of replicates is stated in figure legends.
Randomization	Animals of the indicated ages were randomized in the described groups. For all in vitro experiments, experimental treatment group allocation was random.
Blinding	Histology analyses were performed blinded and the investigators were blinded to group allocation during data collection. For other experiments, the investigators were not blinded to group allocation during the data collection, but were blinded to data analysis.

Behavioural & social sciences study design

All studies must disclose on these points even when the disclosure is negative.

Study description	*Briefly describe the study type including whether data are quantitative, qualitative, or mixed-methods (e.g. qualitative cross-sectional, quantitative experimental, mixed-methods case study).*
Research sample	*State the research sample (e.g. Harvard university undergraduates, villagers in rural India) and provide relevant demographic information (e.g. age, sex) and indicate whether the sample is representative. Provide a rationale for the study sample chosen. For studies involving existing datasets, please describe the dataset and source.*
Sampling strategy	*Describe the sampling procedure (e.g. random, snowball, stratified, convenience). Describe the statistical methods that were used to predetermine sample size OR if no sample-size calculation was performed, describe how sample sizes were chosen and provide a rationale for why these sample sizes are sufficient. For qualitative data, please indicate whether data saturation was considered, and what criteria were used to decide that no further sampling was needed.*
Data collection	*Provide details about the data collection procedure, including the instruments or devices used to record the data (e.g. pen and paper, computer, eye tracker, video or audio equipment) whether anyone was present besides the participant(s) and the researcher, and*

	whether the researcher was blind to experimental condition and/or the study hypothesis during data collection.
Timing	*Indicate the start and stop dates of data collection. If there is a gap between collection periods, state the dates for each sample cohort.*
Data exclusions	*If no data were excluded from the analyses, state so OR if data were excluded, provide the exact number of exclusions and the rationale behind them, indicating whether exclusion criteria were pre-established.*
Non-participation	*State how many participants dropped out/declined participation and the reason(s) given OR provide response rate OR state that no participants dropped out/declined participation.*
Randomization	*If participants were not allocated into experimental groups, state so OR describe how participants were allocated to groups, and if allocation was not random, describe how covariates were controlled.*

Ecological, evolutionary & environmental sciences study design

All studies must disclose on these points even when the disclosure is negative.

Study description	*Briefly describe the study. For quantitative data include treatment factors and interactions, design structure (e.g. factorial, nested, hierarchical), nature and number of experimental units and replicates.*
Research sample	*Describe the research sample (e.g. a group of tagged Passer domesticus, all Stenocereus thurberi within Organ Pipe Cactus National Monument), and provide a rationale for the sample choice. When relevant, describe the organism taxa, source, sex, age range and any manipulations. State what population the sample is meant to represent when applicable. For studies involving existing datasets, describe the data and its source.*
Sampling strategy	*Note the sampling procedure. Describe the statistical methods that were used to predetermine sample size OR if no sample-size calculation was performed, describe how sample sizes were chosen and provide a rationale for why these sample sizes are sufficient.*
Data collection	*Describe the data collection procedure, including who recorded the data and how.*
Timing and spatial scale	*Indicate the start and stop dates of data collection, noting the frequency and periodicity of sampling and providing a rationale for these choices. If there is a gap between collection periods, state the dates for each sample cohort. Specify the spatial scale from which the data are taken*
Data exclusions	*If no data were excluded from the analyses, state so OR if data were excluded, describe the exclusions and the rationale behind them, indicating whether exclusion criteria were pre-established.*
Reproducibility	*Describe the measures taken to verify the reproducibility of experimental findings. For each experiment, note whether any attempts to repeat the experiment failed OR state that all attempts to repeat the experiment were successful.*
Randomization	*Describe how samples/organisms/participants were allocated into groups. If allocation was not random, describe how covariates were controlled. If this is not relevant to your study, explain why.*
Blinding	*Describe the extent of blinding used during data acquisition and analysis. If blinding was not possible, describe why OR explain why blinding was not relevant to your study.*

Did the study involve field work? ☐ Yes ☐ No

Field work, collection and transport

Field conditions	*Describe the study conditions for field work, providing relevant parameters (e.g. temperature, rainfall).*
Location	*State the location of the sampling or experiment, providing relevant parameters (e.g. latitude and longitude, elevation, water depth).*
Access & import/export	*Describe the efforts you have made to access habitats and to collect and import/export your samples in a responsible manner and in compliance with local, national and international laws, noting any permits that were obtained (give the name of the issuing authority, the date of issue, and any identifying information).*
Disturbance	*Describe any disturbance caused by the study and how it was minimized.*

Reporting for specific materials, systems and methods

We require information from authors about some types of materials, experimental systems and methods used in many studies. Here, indicate whether each material, system or method listed is relevant to your study. If you are not sure if a list item applies to your research, read the appropriate section before selecting a response.

Materials & experimental systems

n/a	Involved in the study	
	☒	Antibodies
☒	☐	Eukaryotic cell lines
☒	☐	Palaeontology and archaeology
	☒	Animals and other organisms
☒	☐	Clinical data
☒	☐	Dual use research of concern
☒	☐	Plants

Methods

n/a	Involved in the study	
☒	☐	ChIP-seq
☐	☒	Flow cytometry
☒	☐	MRI-based neuroimaging

Antibodies

Antibodies used

IL-6 Polyclonal antibody, 21865-1-AP, Proteintech, 1/200 dilution
F4/80 Polyclonal antibody, 28463-1-AP, Proteintech, 1/50 dilution
Occludin Polyclonal antibody, 13409-1-AP, Proteintech, 1/200 dilution
MUC2 Polyclonal antibody, 27675-1-AP, Proteintech, 1/200 dilution
Occludin Polyclonal antibody, 27260-1-AP, Proteintech, 1/3000 dilution
Beta Actin Monoclonal antibody, 66009-1-Ig, Proteintech, 1/50000 dilution
IDO1 Rabbit Polyclonal Antibody, AF7161, Beyotime, 1/1000 dilution
ZO-1 Polyclonal antibody, 21773-1-AP, Proteintech, 1/5000 dilution
GAPDH Monoclonal antibody, 60004-1-Ig, Proteintech, 1/100000 dilution
IL-6 rabbit polyclonal antibody, GB11117-100, Servicebio, 1/500 dilution
IL-1 beta rabbit polyclonal antibody, GB11113-100, Servicebio, 1/800 dilution
Histone H3 Mouse Monoclonal Antibody, AF0009, Beyotime, 1/1000 dilution
AHR Rabbit Polyclonal Antibody, AF6165, Beyotime, 1/200 dilution
β-Tubulin Mouse Monoclonal Antibody, AF2835, Beyotime, 1/1000 dilution
TNF alpha Polyclonal Antibody, E-AB-33121, Elabscience, 1/600 dilution
Brilliant Violet 421 anti-human CD11c Antibody, Biolegend, 301628

Flow cytometry
Viability Dye (564995, BD Horizon, 1:100)
anti-CD3 (11-0032-82, eBioscience, 1:150)
anti-CD4 (48-0041-82, eBioscience, 1:150)
anti-CD25 (17-0251-82, eBioscience, 1:150)
anti-FOXP3 (12-5773-82, eBioscience, 1:75)
anti-IL17 (45-7177-82, eBioscience, 1:75)
anti-IFNy (505808, Biolegend, 1:75)

Validation

IL-6 Polyclonal antibody, Xiao Y, 2021, Cancer Cell, 39(3):423-437
F4/80 Polyclonal antibody, Pan T, 2021, Theranostics, 11(3):1192-1206
Occludin Polyclonal antibody, Zhao M, 2018, EMBO Mol Med, 10(8):e8736
MUC2 Polyclonal antibody, Bai R, 2020, EMBO J, 39(13):e103325
Occludin Polyclonal antibody, Zhao Z, 2021, Acta Pharm Sin B, 11(9):2859-2879
Beta Actin Monoclonal antibody, Qian H, 2020, Nature, 582(7813):550-556
ZO-1 Polyclonal antibody, Wang Z, 2023, Redox Biol, 60:102618
GAPDH Monoclonal antibody, Zhou R, 2022, Nature, 612(7940):519-527
IL-6 rabbit polyclonal antibody, Deng Z, 2020, Oxid Med Cell Longev, 2020:4964202
IL-1 beta rabbit polyclonal antibody, Yang L, 2022, Food Sci Nutr, 10(5):1357-1367
Histone H3 Mouse Monoclonal Antibody, Xu D, 2020, J Pineal Res, 69(4):e12690
TNF alpha Polyclonal Antibody, Fischer A, 2022, 23(4):518-531

Palaeontology and Archaeology

Specimen provenance	*Provide provenance information for specimens and describe permits that were obtained for the work (including the name of the issuing authority, the date of issue, and any identifying information). Permits should encompass collection and, where applicable, export.*
Specimen deposition	*Indicate where the specimens have been deposited to permit free access by other researchers.*
Dating methods	*If new dates are provided, describe how they were obtained (e.g. collection, storage, sample pretreatment and measurement), where they were obtained (i.e. lab name), the calibration program and the protocol for quality assurance OR state that no new dates are provided.*

☐ Tick this box to confirm that the raw and calibrated dates are available in the paper or in Supplementary Information.

Ethics oversight	*Identify the organization(s) that approved or provided guidance on the study protocol, OR state that no ethical approval or guidance was required and explain why not.*

Note that full information on the approval of the study protocol must also be provided in the manuscript.

Animals and other research organisms

Policy information about studies involving animals; ARRIVE guidelines recommended for reporting animal research, and Sex and Gender in Research

Laboratory animals	Six- to eight-week-old and sex matched mice on C57BL/6J background were used in this study. Germ-free (GF) C57BL/6J mice were bred and housed at the Shenzhen Gnotobio Biotechnology Co., Ltd. GF status was confirmed through 16S qPCR analysis before used for relative experiments, which were also carried out at Shenzhen Gnotobio Biotechnology Co., Ltd. Wild-type C57BL/6J (WT) mice were purchased from the Model Animal Research Center of Nanjing University (Nanjing, China). Foxp3-DTR mice were a gift from Dr. Bin Li from Department of Immunology and Microbiology, Shanghai Institute of Immunology, Shanghai Jiao Tong University School of Medicine. Foxp3-DTR mice were given intraperitoneal (i.p.) injections of 1 mg DTx (50 ng DT/g body weight) once per day for 7 consecutive days to guarantee successful depletion of Tregs. Splenocytes were analyzed to verify the elimination efficiency of Tregs by flow cytometry. Indoleamine-2,3-dioxygenase 1 knockout mice (Ido1-/-) were kindly provided by Dr. Yajing Wang from State Key Laboratory of Natural Medicines, Department of Physiology, China Pharmaceutical University. G-protein coupled receptor 43 knockout mice (Gpr43-/-) and IL-17-EGFP transgenic mice were purchased from Cyagen Biosciences Inc (Suzhou, China). The mice were routinely maintained in a specific-pathogen-free facility with a temperature- and humidity-controlled environment (22 ± 2 °C, 50 ± 10% humidity), and under a constant 12 h light/dark cycle, and were given free access to a regular chow diet (Gat# P1101F-25, Shanghai SLACOM) and water throughout study at Zhejiang University. All procedures were conducted in compliance with a protocol approved by the IACUC at Zhejiang University, China.<br>Mice were given ampicillin (Amp 10 mg), neomycin (Neo 10 mg), metronidazole (Metro 10 mg), or vancomycin (Van), individually or in combination (referred to as Abx) daily for 5 days via oral gavage. Fecal samples collected from microbiota-depleted mice at the 5th day post-treatment were homogenized, plated on BHI agar with 10% sheep blood, and cultured under anaerobic conditions at 37 °C for 2 days followed by incubation under aerobic conditions at 37 °C for 1 day to confirm efficient microbial depletion.
Wild animals	No wild animals were used in this study.
Reporting on sex	The research process with the method of male and female in half, so the study not only applies to one gender.
Field-collected samples	No field-collected samples were used in this study.
Ethics oversight	All animal experiments were strictly carried out in accordance with protocols approved (No.117113) by the Institutional Animal Carel and Use Committee of Zhejiang University.

Note that full information on the approval of the study protocol must also be provided in the manuscript.

Clinical data

Policy information about clinical studies
All manuscripts should comply with the ICMJEguidelines for publication of clinical research and a completedCONSORT checklist must be included with all submissions.

Clinical trial registration	*Provide the trial registration number from ClinicalTrials.gov or an equivalent agency.*
Study protocol	*Note where the full trial protocol can be accessed OR if not available, explain why.*
Data collection	*Describe the settings and locales of data collection, noting the time periods of recruitment and data collection.*
Outcomes	*Describe how you pre-defined primary and secondary outcome measures and how you assessed these measures.*

Dual use research of concern

Policy information about dual use research of concern

Hazards

Could the accidental, deliberate or reckless misuse of agents or technologies generated in the work, or the application of information presented in the manuscript, pose a threat to:

No	Yes	
☐	☐	Public health
☐	☐	National security
☐	☐	Crops and/or livestock
☐	☐	Ecosystems
☐	☐	Any other significant area

Experiments of concern

Does the work involve any of these experiments of concern:

No	Yes	
☐	☐	Demonstrate how to render a vaccine ineffective
☐	☐	Confer resistance to therapeutically useful antibiotics or antiviral agents
☐	☐	Enhance the virulence of a pathogen or render a nonpathogen virulent
☐	☐	Increase transmissibility of a pathogen
☐	☐	Alter the host range of a pathogen
☐	☐	Enable evasion of diagnostic/detection modalities
☐	☐	Enable the weaponization of a biological agent or toxin
☐	☐	Any other potentially harmful combination of experiments and agents

Plants

Seed stocks	*Report on the source of all seed stocks or other plant material used. If applicable, state the seed stock centre and catalogue number. If plant specimens were collected from the field, describe the collection location, date and sampling procedures.*
Novel plant genotypes	*Describe the methods by which all novel plant genotypes were produced. This includes those generated by transgenic approaches, gene editing, chemical/radiation-based mutagenesis and hybridization. For transgenic lines, describe the transformation method, the number of independent lines analyzed and the generation upon which experiments were performed. For gene-edited lines, describe the editor used, the endogenous sequence targeted for editing, the targeting guide RNA sequence (if applicable) and how the editor was applied.*
Authentication	*Describe any authentication procedures for each seed stock used or novel genotype generated. Describe any experiments used to assess the effect of a mutation and, where applicable, how potential secondary effects (e.g. second site T-DNA insertions, mosiacism, off-target gene editing) were examined.*

ChIP-seq

Data deposition

☐ Confirm that both raw and final processed data have been deposited in a public database such as GEO.

☐ Confirm that you have deposited or provided access to graph files (e.g. BED files) for the called peaks.

Data access links<br>*May remain private before publication.*	*For "Initial submission" or "Revised version" documents, provide reviewer access links. For your "Final submission" document, provide a link to the deposited data.*
Files in database submission	*Provide a list of all files available in the database submission.*
Genome browser session<br>(e.g. UCSC)	*Provide a link to an anonymized genome browser session for "Initial submission" and "Revised version" documents only, to enable peer review. Write "no longer applicable" for "Final submission" documents.*

Methodology

Replicates	*Describe the experimental replicates, specifying number, type and replicate agreement.*
Sequencing depth	*Describe the sequencing depth for each experiment, providing the total number of reads, uniquely mapped reads, length of reads and whether they were paired- or single-end.*
Antibodies	*Describe the antibodies used for the ChIP-seq experiments; as applicable, provide supplier name, catalog number, clone name, and lot number.*
Peak calling parameters	*Specify the command line program and parameters used for read mapping and peak calling, including the ChIP, control and index files used.*
Data quality	*Describe the methods used to ensure data quality in full detail, including how many peaks are at FDR 5% and above 5-fold enrichment.*
Software	*Describe the software used to collect and analyze the ChIP-seq data. For custom code that has been deposited into a community repository, provide accession details.*

Flow Cytometry

Plots

Confirm that:

- [x] The axis labels state the marker and fluorochrome used (e.g. CD4-FITC).
- [x] The axis scales are clearly visible. Include numbers along axes only for bottom left plot of group (a 'group' is an analysis of identical markers).
- [x] All plots are contour plots with outliers or pseudocolor plots.
- [x] A numerical value for number of cells or percentage (with statistics) is provided.

Methodology

Sample preparation	Murine IECs and LPMCs were obtained from the colon as previously described. Briefly, the colon was opened longitudinally and cut into pieces. After incubation with EDTA (5.5 mM) and dithiothreitol (DTT) (1 mM) in Hank's balanced salt solution (HBSS), vortexing and passing through a 70-µm cell strainer, the suspension of IECs was washed twice by centrifugation at 100 x g for 2 min and collected for future experiments. The remaining lamina propria tissue was incubated with digestion solution containing collagenase (1 mg/mL) and DNase (0.2 mg/mL). The resulting LPMC cell suspension was subjected to Percoll-gradient separation and harvested for further experiments. For cell surface staining, single cell suspensions were incubated on ice for 30 min with the following antibodies: FITC-conjugated anti-CD3 (11-0032-82, eBioscience), eFluor 450-conjugated anti-CD4 (48-0041-82, eBioscience), and APC-conjugated anti-CD25 (17-0251-82, eBioscience).For Treg cell analysis after staining of cells with CD3, CD4 and CD25 antibodies, lymphocyte suspensions were fixed and permeabilized using transcription factor buffer sets (562574, BD Pharmingen) according to the manufacturer's instructions and stained with anti-Foxp3-PE (12-5773-82, eBioscience). For analysis of Th1 and Th17 cells, isolated tissue lymphocytes were stimulated for 5 h with cell stimulation cocktail plus protein transport inhibitors (00-4975-93, eBioscience). After incubation for 5 h, cells were washed in PBS and stained for cell death using fixable viability stain 570 (564995, BD Horizon), FITC-anti-CD3, and eFluor 450-anti-CD4. Stained cells were fixed in fixation buffer (00-8222-49, eBioscience), permeabilized with intracellular staining permeabilization wash buffer (00-8333-56, eBioscience), and stained with anti-IL-17A conjugated to PerCP-Cyanine5.5 (45-7177-82, eBioscience) and phycoerythrin-conjugated anti-IFN-γ (505808, Biolegend)
Instrument	BD FACSVerse
Software	FlowJo V10
Cell population abundance	Abundance is reported in figures and methods where relevant.
Gating strategy	Gating strategies are specified within the text or figure legend of supplementary figure 5 for relevant flow cytometry experiments.

- [x] Tick this box to confirm that a figure exemplifying the gating strategy is provided in the Supplementary Information.

Magnetic resonance imaging

Experimental design

Design type	*Indicate task or resting state; event-related or block design.*
Design specifications	*Specify the number of blocks, trials or experimental units per session and/or subject, and specify the length of each trial or block (if trials are blocked) and interval between trials.*

Behavioral performance measures | *State number and/or type of variables recorded (e.g. correct button press, response time) and what statistics were used to establish that the subjects were performing the task as expected (e.g. mean, range, and/or standard deviation across subjects).*

Acquisition

Imaging type(s) | *Specify: functional, structural, diffusion, perfusion.*

Field strength | *Specify in Tesla*

Sequence & imaging parameters | *Specify the pulse sequence type (gradient echo, spin echo, etc.), imaging type (EPI, spiral, etc.), field of view, matrix size, slice thickness, orientation and TE/TR/flip angle.*

Area of acquisition | *State whether a whole brain scan was used OR define the area of acquisition, describing how the region was determined.*

Diffusion MRI ☐ Used ☐ Not used

Preprocessing

Preprocessing software | *Provide detail on software version and revision number and on specific parameters (model/functions, brain extraction, segmentation, smoothing kernel size, etc.).*

Normalization | *If data were normalized/standardized, describe the approach(es): specify linear or non-linear and define image types used for transformation OR indicate that data were not normalized and explain rationale for lack of normalization.*

Normalization template | *Describe the template used for normalization/transformation, specifying subject space or group standardized space (e.g. original Talairach, MNI305, ICBM152) OR indicate that the data were not normalized.*

Noise and artifact removal | *Describe your procedure(s) for artifact and structured noise removal, specifying motion parameters, tissue signals and physiological signals (heart rate, respiration).*

Volume censoring | *Define your software and/or method and criteria for volume censoring, and state the extent of such censoring.*

Statistical modeling & inference

Model type and settings | *Specify type (mass univariate, multivariate, RSA, predictive, etc.) and describe essential details of the model at the first and second levels (e.g. fixed, random or mixed effects; drift or auto-correlation).*

Effect(s) tested | *Define precise effect in terms of the task or stimulus conditions instead of psychological concepts and indicate whether ANOVA or factorial designs were used.*

Specify type of analysis: ☐ Whole brain ☐ ROI-based ☐ Both

Statistic type for inference | *Specify voxel-wise or cluster-wise and report all relevant parameters for cluster-wise methods.*

(See Eklund et al. 2016)

Correction | *Describe the type of correction and how it is obtained for multiple comparisons (e.g. FWE, FDR, permutation or Monte Carlo).*

Models & analysis

n/a	Involved in the study
☐	☐ Functional and/or effective connectivity
☐	☐ Graph analysis
☐	☐ Multivariate modeling or predictive analysis

Functional and/or effective connectivity | *Report the measures of dependence used and the model details (e.g. Pearson correlation, partial correlation, mutual information).*

Graph analysis | *Report the dependent variable and connectivity measure, specifying weighted graph or binarized graph, subject- or group-level, and the global and/or node summaries used (e.g. clustering coefficient, efficiency, etc.).*

Multivariate modeling and predictive analysis | *Specify independent variables, features extraction and dimension reduction, model, training and evaluation metrics.*
