# Supplementary material for: Dubosiella newyorkensis modulates immune tolerance in colitis via the L-lysine-activated AhR-IDO1-Kyn pathway
Source: Nat Commun. 2024 Feb 13;15:1333. doi: 10.1038/s41467-024-45636-x (PMC10864277; doi:10.1038/s41467-024-45636-x)

Fig. 2d

ZO-1

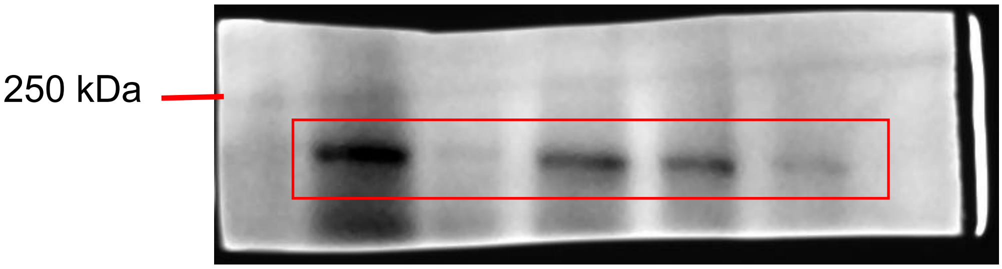

Occludin

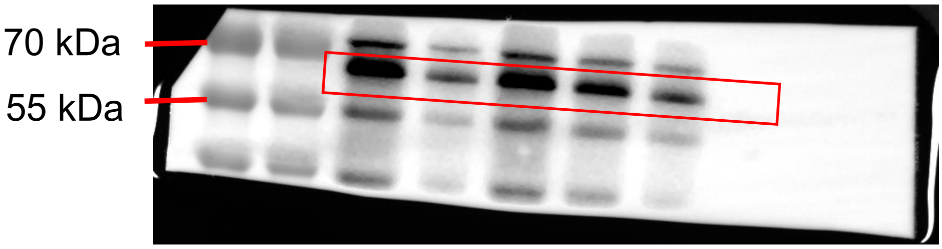

GAPDH

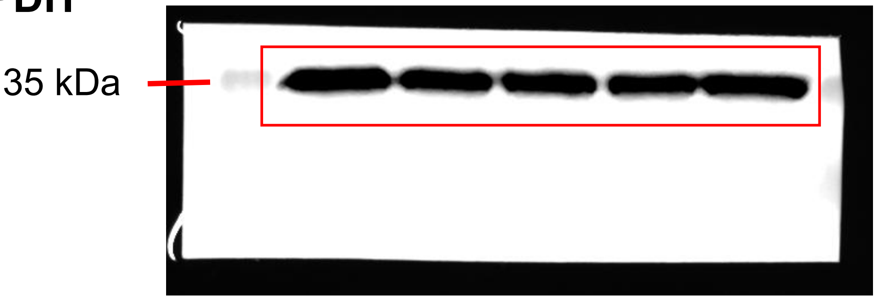

Fig. 2I

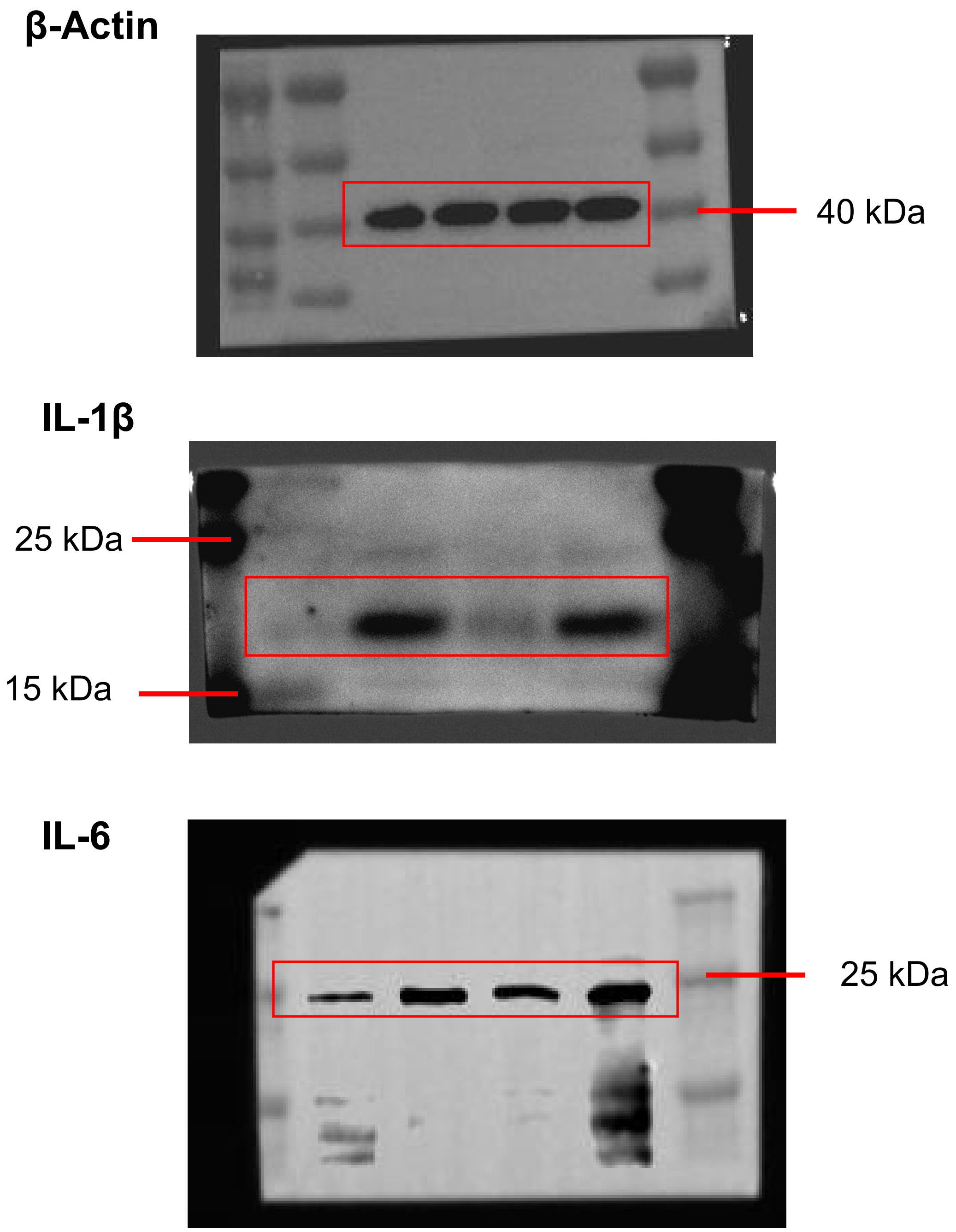

Fig. 2I

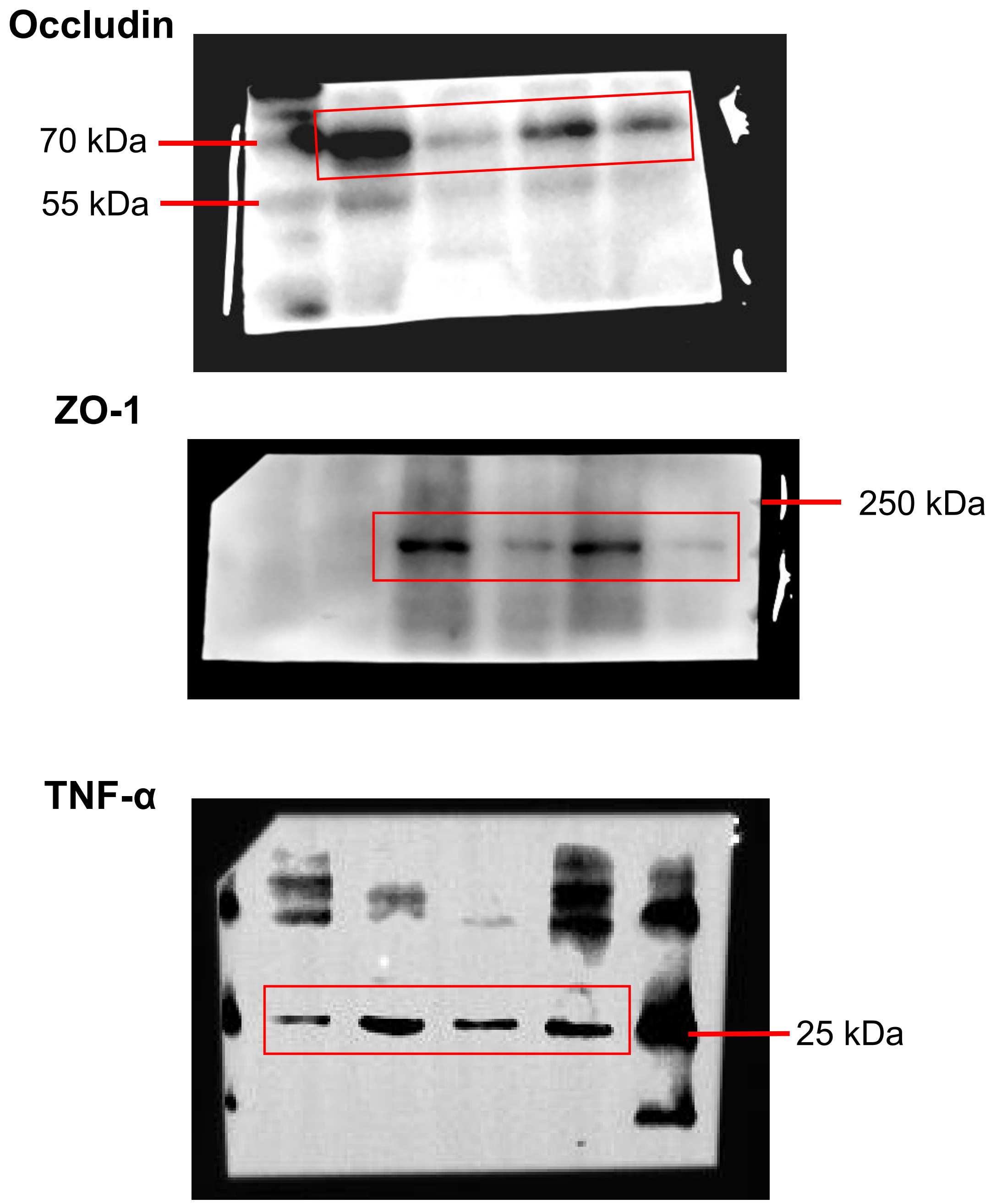

Fig. 4h

GAPDH

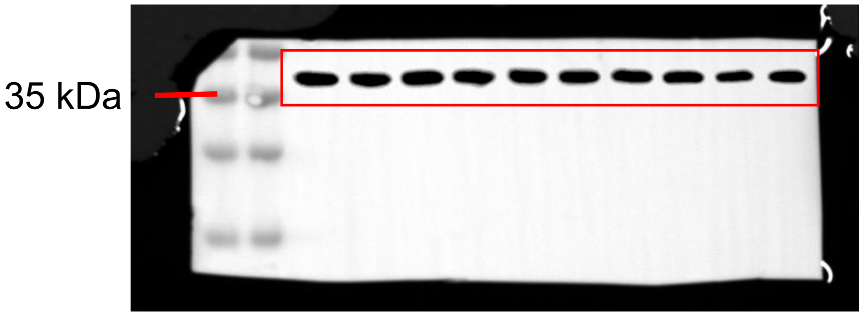

IDO1

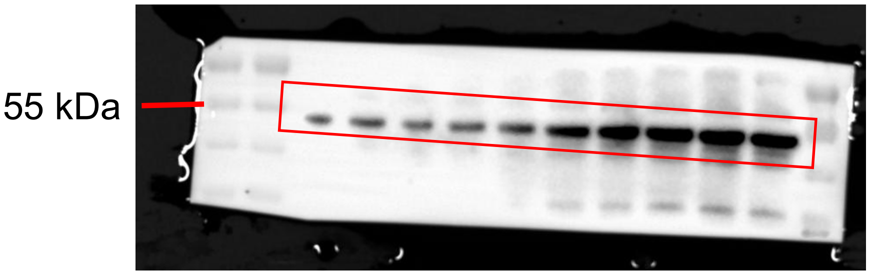

Fig. 5c

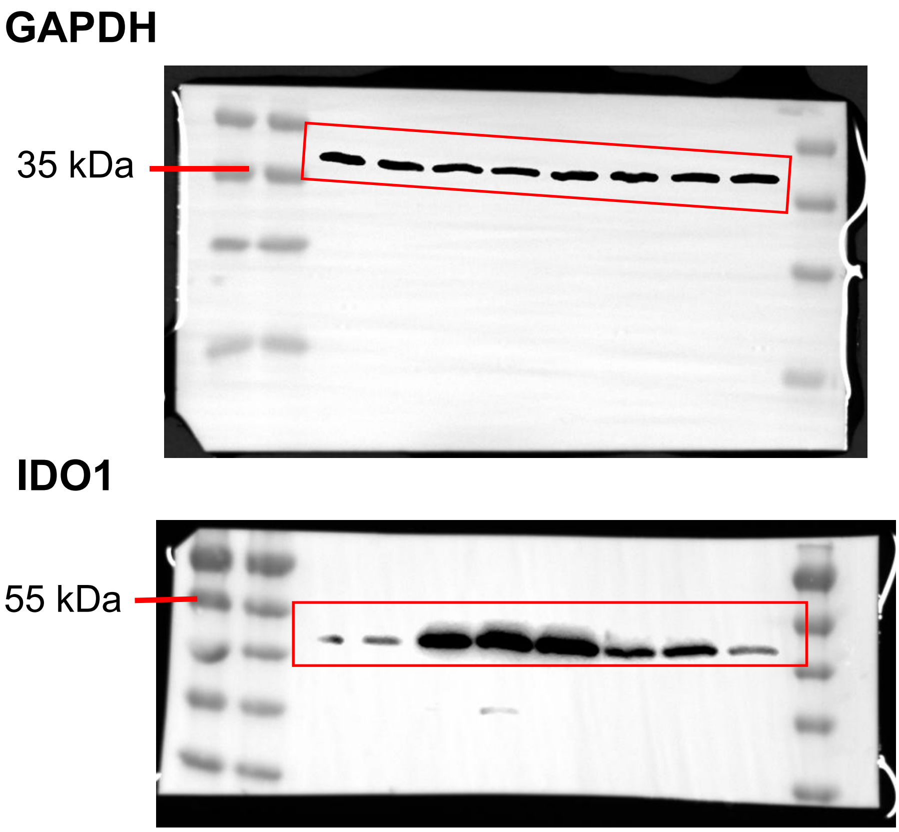

Fig. 6e

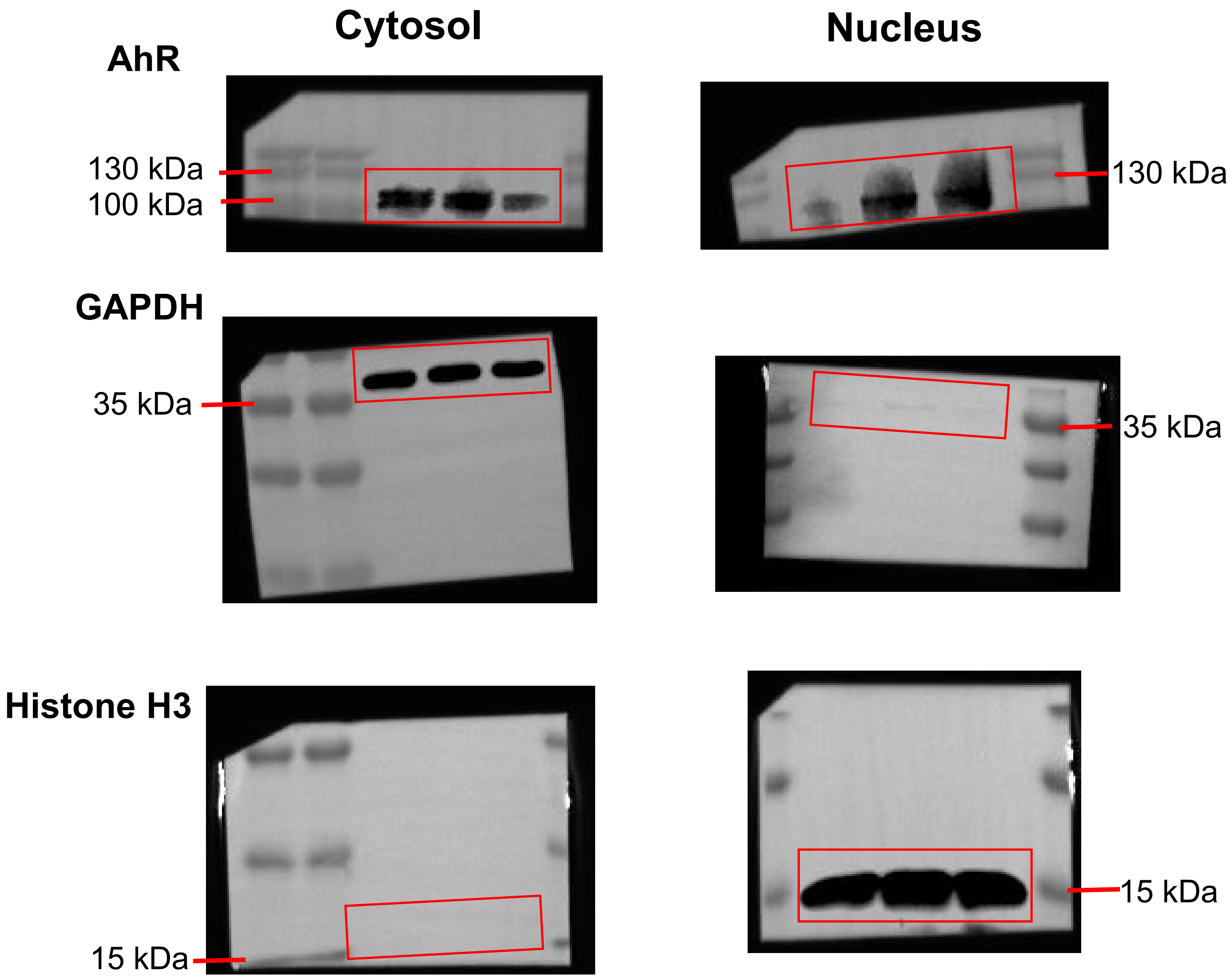
Cytosol
Nucleus
AhR
130 kDa
100 kDa
130 kDa
GAPDH
35 kDa
35 kDa
Histone H3
15 kDa
15 kDa

Supplementary Fig. 6i

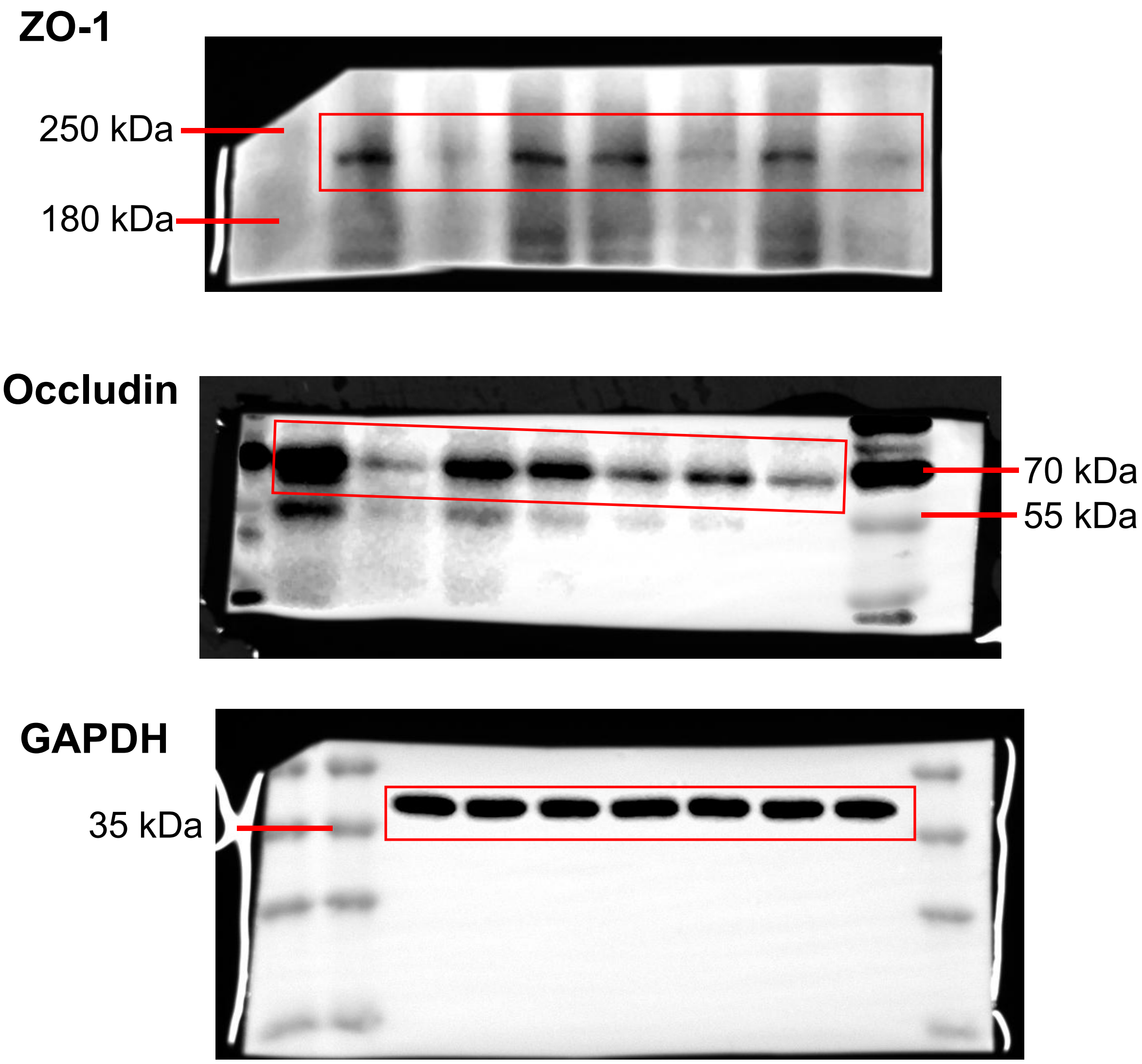

Supplementary Fig. 8c

GAPDH

35 kDa

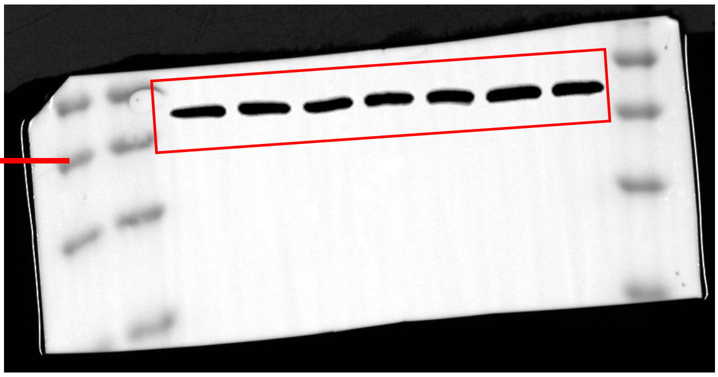

IL-1β

25 kDa

15 kDa

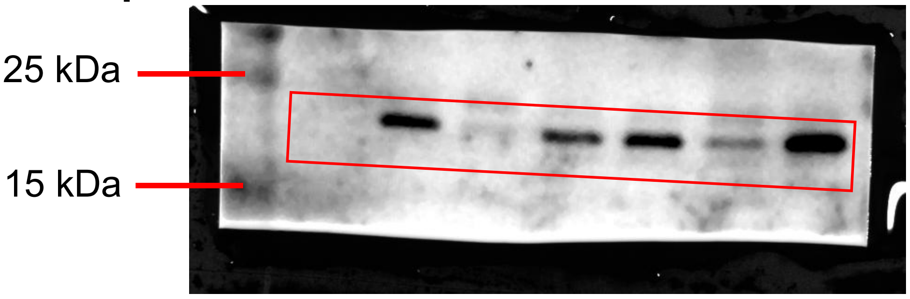

IL-6

25 kDa

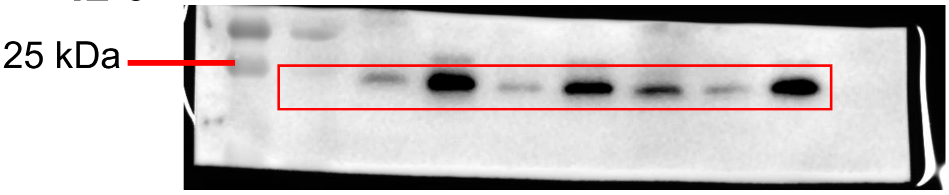

Supplementary Fig. 8c

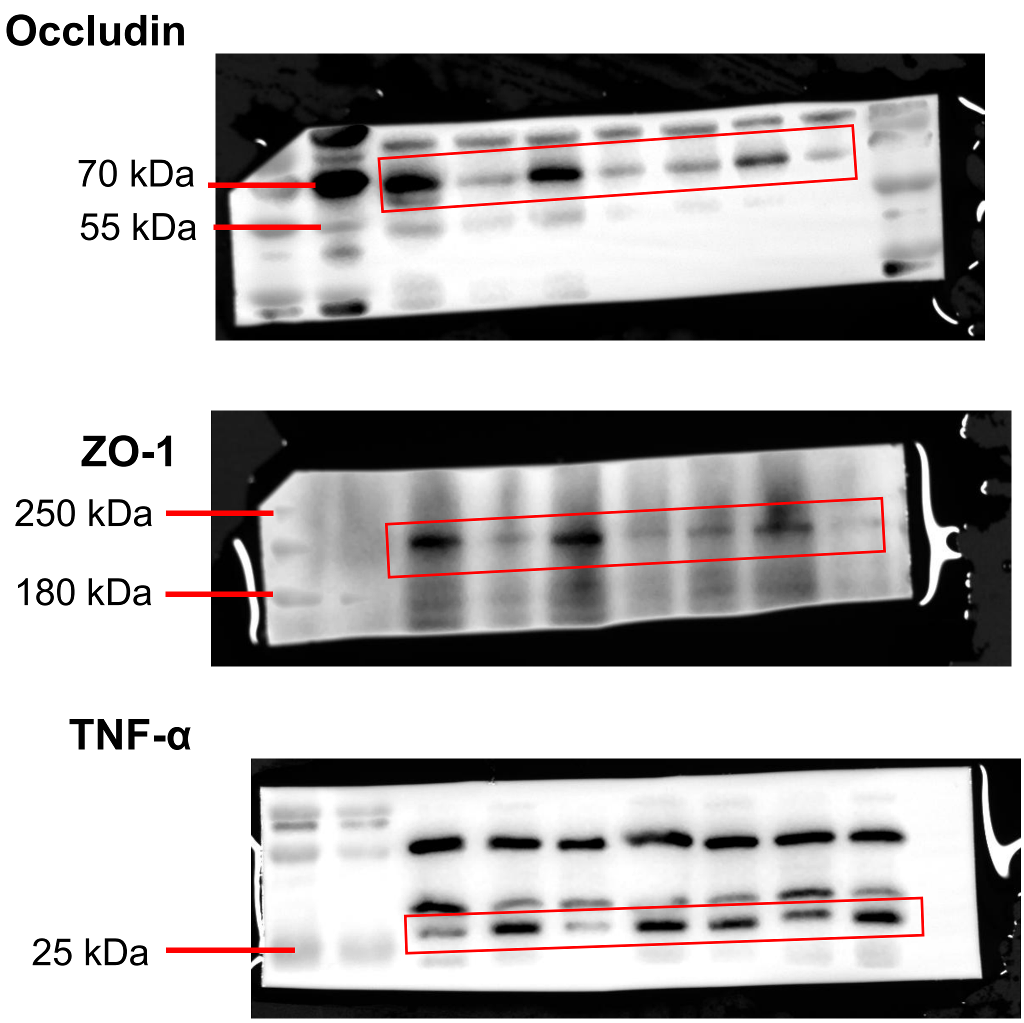

Supplement: Supplementary file 4 — Source Data [file 41467_2024_45636_MOESM4_ESM.zip › Source_Data_Unprocessed_western_blots.pdf]
